# The peroxisome: an update on mysteries 2.0

**DOI:** 10.1007/s00418-018-1722-5

**Published:** 2018-09-15

**Authors:** Markus Islinger, Alfred Voelkl, H. Dariush Fahimi, Michael Schrader

**Affiliations:** 1 Institute of Neuroanatomy, Center for Biomedicine and Medical Technology Mannheim, Medical Faculty Manheim, University of Heidelberg, 68167 Mannheim, Germany; 2 Institute for Anatomy and Cell Biology, University of Heidelberg, 69120 Heidelberg, Germany; 3 Biosciences, University of Exeter, Exeter, EX4 4QD UK

**Keywords:** ACBD5, Alzheimer, Cancer, Hearing loss, Membrane contact sites, Motility, Multiple sclerosis, Organelle biogenesis, Organelle division, Organelle dynamics, Parkinson, Peroxin, Peroxisome

## Abstract

Peroxisomes are key metabolic organelles, which contribute to cellular lipid metabolism, e.g. the β-oxidation of fatty acids and the synthesis of myelin sheath lipids, as well as cellular redox balance. Peroxisomal dysfunction has been linked to severe metabolic disorders in man, but peroxisomes are now also recognized as protective organelles with a wider significance in human health and potential impact on a large number of globally important human diseases such as neurodegeneration, obesity, cancer, and age-related disorders. Therefore, the interest in peroxisomes and their physiological functions has significantly increased in recent years. In this review, we intend to highlight recent discoveries, advancements and trends in peroxisome research, and present an update as well as a continuation of two former review articles addressing the unsolved mysteries of this astonishing organelle. We summarize novel findings on the biological functions of peroxisomes, their biogenesis, formation, membrane dynamics and division, as well as on peroxisome–organelle contacts and cooperation. Furthermore, novel peroxisomal proteins and machineries at the peroxisomal membrane are discussed. Finally, we address recent findings on the role of peroxisomes in the brain, in neurological disorders, and in the development of cancer.

## Introduction

The interest in peroxisomes and their (patho)physiological roles in health and disease is constantly increasing within the scientific community, and there is no doubt that peroxisomes are on the rise. Since their discovery more than 60 years ago (Rhodin 1954), essential metabolic functions of peroxisomes [e.g. in lipid metabolism including fatty acid β-oxidation and synthesis of myelin sheath lipids, or metabolism of reactive oxygen species (ROS), in particular, hydrogen peroxide] have been revealed, demonstrating that peroxisomes are key metabolic organelles, and their dysfunction has been linked to severe metabolic disorders in man. In recent years, it became clear that peroxisomes also fulfil crucial non-metabolic roles, e.g. in cellular stress responses, the combat of pathogens and antiviral defence, as cellular signalling platforms and in healthy ageing. These findings indicate that peroxisomes are also “protective” organelles with a wider significance in human health and potential impact on a large number of globally important human diseases such as neurodegeneration, obesity, cancer, and age-related disorders. However, many physiological roles of peroxisome still remain enigmatic. Here, we will highlight recent discoveries, advancements and trends in peroxisome research, which, we hope, will also aid non-experts and those who are not up to date with the current developments to get an overview of the field of peroxisome biology. This review represents an update as well as a continuation of two articles of our “mystery” series we published in *Histochemistry and Cell Biology* [the 1st on the occasion of the 50th anniversary of the journal in 2008 (Schrader and Fahimi 2008; Islinger et al. 2012a, b)]. To avoid repetition, we will refer to those articles when appropriate and to more specialized recent reviews on peroxisome biology. New advances in the understanding of pexophagy, the controlled removal of peroxisomes, are addressed by Kovacs and coworkers (see this issue) (Eberhart and Kovacs 2018).

## Mysterious functions: an update on peroxisomal metabolism

### An organelle—underrated at the beginning—hesitantly discloses its mysteries

The subcellular structure delineated by a single membrane surrounding a granular homogeneous matrix, discovered in rodent kidney cells and subsequently in liver, and termed “microbody” to meet its morphology (Rhodin 1954; Rouiller and Bernhard 1956), initially had the standing of a cell oddity with no clear role in vital functions and intermediary metabolism. In the succeeding decades, however, evidence accumulated progressively converting the obscure “Cinderella” amongst the known cell organelles to a multifunctional global player with profound and far-reaching relevance for health and disease of animal and plant organisms.

Initiated by the pioneering work of De Duve`s group with the clear-cut biochemical individualisation and characterization of microbodies—since then renamed *peroxisomes*—(De Duve 1965; De Duve and Baudhuin 1966; see Vamecq et al. 2014 for further ref.), and the observation that peroxisomes are lacking in Zellweger patients (Goldfischer et al. 1973), it is now well documented that peroxisomes are indispensable to eukaryotic cells and hence virtually ubiquitously distributed. They are unique in their morphological heterogeneity (see “Peroxisome heterogeneity”) and display a remarkable functional plasticity in both anabolic and catabolic processes, specifically adapted in their proteome to cell type, growth conditions and variable environment (see “Mysterious machinery: new proteins and functions at the peroxisomal membrane”). Last but not least, they are involved in fundamental vital processes such as the detoxification of dangerous oxygen/nitrogen species (Fransen et al. 2012), are signalling platforms (Mast et al. 2015), with critical roles for innate immunity (Dixit et al. 2010) as well as development and differentiation (Titorenko and Rachubinski 2004), and intimately communicate with other organelles (Schrader et al. 2013; Shai et al. 2016). Dysfunctions or even lack of peroxisomes not only underlie the well-known peroxisomal disorders, but also contribute to physio- as well as patho-physiological processes such as ageing and related diseases (Deori et al. 2018) or cancer (see “Peroxisomes and cancer: a mysterious connection”).

To contribute in concert to the well-being of a cell, and to optimize their multiple functions, peroxisomes collaborate and communicate with other cell organelles. Numerous mechanisms have evolved enabling such a crosstalk including signal transduction pathways, vesicular trafficking and contact sites (see “Peroxisome–organelle interactions: the mysterious world of tethers”; Shai et al. 2016). Crosstalk between peroxisomes, the ER and the mitochondria is the most common, yet neither the underlying mechanisms nor the functional relevance is experimentally verified in great detail (Shai et al. 2016 and ref. therein).

The impact peroxisomes evidently have on lipid metabolism is best documented by the accumulation of very long chain fatty acids (VLCFA) in plasma, and the complete deficiency of plasmalogens in tissues of Zellweger patients (Brown et al. 1982; Heymans et al. 1983). Ether lipid synthesis occurs in peroxisomes and begins with the esterification of dihydroxyacetone phosphate (DHAP) with a long-chain fatty acid by the enzyme DHAP acyltransferase (DHAPAT), and the subsequent replacement of the fatty acid by a fatty alcohol to form alkyl-DHAP by alkyl-glycerone phosphate synthase (AGPS). Remarkably, the critical AGPS enzyme is heightened in aggressive cancer cells and primary human breast tumors, and its genetic ablation significantly impairs cancer aggressiveness and tumorigenesis (Benjamin et al. 2013). Since it could be demonstrated that AGPS knockdown had dramatic effects upon tumor growth in mice, and inhibition of AGPS activity lowers ether lipids and impairs cancer pathogenicity in different types of human cancer cells (Piano et al. 2015), the development of efficacious appropriate inhibitors might be crucial in cancer therapy.

Peroxisomes and mitochondria interact intensively, inter alia, in fatty acid (Wanders 2014), as well as ROS metabolism (Fransen et al. 2012; Lismont et al. 2015), and in the detoxification of glyoxylate and phytanic acid (Wanders et al. 2011). Most importantly, peroxisomes exclusively β-oxidize VLCFA. Increased concentrations of VLCFA are found in body fluids and tissues of patients with X-ALD as well as acyl-CoA oxidase 1 (ACOX1) deficiency, affecting in particular the nervous system (Wanders et al. 2010 and ref. therein). Appropriate cytotoxic properties of VLCFA reported include inflammatory demyelination and axonopathy, cell death of oligodendrocytes and astrocytes, deregulation of intracellular Ca^2+^ homeostasis, and a marked decrease of the membrane potential of mitochondria in oligodendrocytes (Hein et al. 2008; see also “News from the brain: unravelling the mysterious role of peroxisomes in the central nervous system (CNS)”).

In mammalian organisms including humans, α-oxidation of 3-methyl-branched-chain fatty acids such as phytanic acid is a strictly peroxisomal process. To explain the toxic properties of phytanic acid when not properly processed, it was initially hypothesized that its incorporation into membranes disrupts the arrangement of lipids and their interactions with proteins, hence their integrity. Alternatively, based on the notion that the chemical structure of phytanic acid shows similarities to that of the vitamins A, E, and K, phytanic acid could act as an anti-metabolite with respect to these isoprenoids. Subsequent in vitro studies mainly focused on the effects of phytanic acid on mitochondria (Schönfeld and Struy 1999), yet it remains to be clarified whether these in vitro effects also meet the in vivo situation of Refsum disease.

## Peroxisome heterogeneity

The heterogeneity of peroxisomes was already noted in early electron microscopic studies, when they were still referred to as “microbodies and related particles” (Hruban and Rechcigl 1969). The discovery of hydrogen peroxide metabolism and the designation as “peroxisome” emphasized the similarity and the close relationship of this group of organelles in animal and plant cells (De Duve and Baudhuin 1966). But subsequent studies revealed the characteristic features of peroxisomes of different organs, e.g. the marked differences between peroxisomes from rat liver and brain (Gaunt and de Duve 1976). Moreover, the alterations of enzymes of peroxisomes in the course of pre- and post-natal development revealed the capability of this organelle to adapt to differing metabolic requirements of the organism (Krahling et al. 1979). For a review on the diversity of peroxisomes in the animal kingdom, see Islinger et al. (2010). The heterogeneity of peroxisomes can be clearly demonstrated by the cytochemical technique for D-amino acid oxidase using cerium (Angermüller and Fahimi 1988; Angermüller 1989). In rat hepatocytes, a mosaic pattern with strongly and weakly reactive peroxisomes is observed with overall staining being stronger in peri-portal (high oxygen conc.) than in peri-central (low oxygen conc.) parts of the liver lobule. In the kidney, the proximal tubules of the renal cortex are strongly stained with the rest of the nephron being negative. In particular, in some cells, strongly and weakly stained peroxisomes are present side by side within the same cells (Angermüller and Fahimi 1988). The existence of heterogeneous subpopulations of peroxisomes has also been observed in biochemical studies when peroxisomes were isolated (Lüers et al. 1993; Islinger et al. 2012) and in morphological studies with cultured mammalian cells (Schrader et al. 1994) or during fungal development (Takano-Rojas et al. 2016). These differences have been linked to peroxisome formation and maturation (see “Peroxisome formation: mysterious with a new twist” and “Mysterious multiplication: new insights into peroxisome division”). Interestingly, both, de novo formation of peroxisomes from the ER via pre-peroxisomal vesicles or from pre-existing organelles via membrane growth and division, lead to the formation of membrane compartments which mature by subsequent import of matrix proteins (Hoepfner et al. 2005; Delille et al. 2010). The matrix protein content of pre-existing peroxisomes is therefore not evenly distributed over new organelles indicating that peroxisome formation by division is an asymmetric process (Huybrechts et al. 2009; Delille et al. 2010). Peroxisomes display an age-related heterogeneity with respect to their capacity to incorporate newly synthesized proteins (Huybrechts et al. 2009) and segregation during cell division (Kumar et al. 2018). This also applies to peroxisomal membrane proteins (PMPs), which reorganize in the peroxisomal membrane during membrane growth and division (Delille et al. 2010; Cepińska et al. 2011). The application of super-resolution microscopy supported the notion that PMPs are compartmentalized (Galiani et al. 2016; Soliman et al. 2018). A further degree of heterogeneity (and PMP compartmentalisation) is achieved by the dynamic formation of membrane contact sites with other organelles, e.g. the ER (see “Peroxisome–organelle interactions: the mysterious world of tethers”). Tethering impacts on peroxisome motility and likely explains why only a subset of peroxisomes exhibits long-range movement along cytoskeletal tracks resulting in a heterogeneous motile behaviour (Costello et al. 2017; Castro et al. 2018a, b) (see “Peroxisome motility and distribution: mysterious movers”).

## Mysterious machinery: new proteins and functions at the peroxisomal membrane

### News on peroxins, protein import, molecular mechanisms and membrane adaptors

**Fig. 1:**
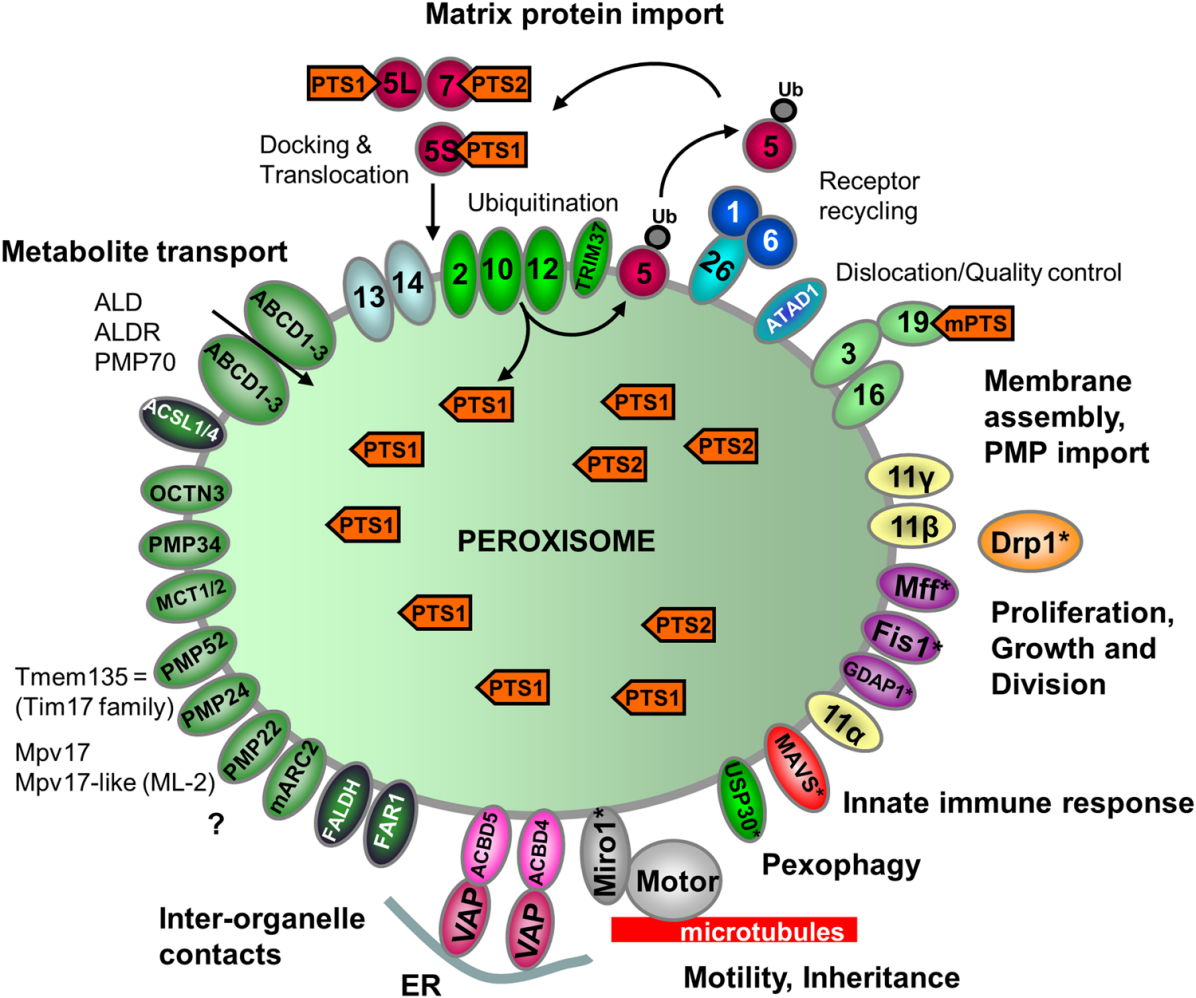
Schematic overview of the molecular machineries and proteins localized at the membranes of peroxisomes in mammals. Adapted from Schrader and Fahimi (2008). See text for further details. Matrix protein import: after synthesis on free ribosomes, cargo proteins containing the peroxisomal targeting signals PTS1 or PTS2 bind to the corresponding cytosolic receptors Pex5 or Pex7 and form receptor–cargo complexes. The Pex7–cargo complex requires accessory factors for import (Pex5pL, a long isoform of Pex5p, in mammals and plants, Pex18p and Pex21p in *S. cerevisiae*, Pex20p in *Neurospora crassa, Yarrowia lipolytica*, and *Hansenula polymorpha*). Pex9 is a new Pex5-like yeast peroxisomal targeting receptor. Import is achieved by a complex set of integral or peripheral PMPs that form the matrix protein import machinery, which mediates docking of the cargo-bound import receptor at the peroxisomal membrane, cargo translocation into the matrix of the organelle by a dynamic translocon, and export of the receptor back to the cytosol. Recycling of the receptor involves its ubiquitination (ub) and extraction from the membrane by an AAA–ATPase complex (Pex1, Pex6). Pex4 is an ubiquitin-conjugating enzyme that is bound to Pex22. Pex6 binds to Pex15 in *S. cerevisiae* or to Pex26 in humans. The DnaJ-like protein Djp1p assists in matrix protein import. Membrane assembly and insertion of PMPs (containing an mPTS) depend on Pex19, Pex3 and Pex16. Pex19 functions as a cycling receptor/chaperone, which binds the PMPs in the cytosol and interacts with Pex3 at the peroxisomal membrane. Yeast Pex36 is a new functional homolog of mammalian Pex16. Proliferation, growth and division: Pex11α, Pex11β and Pex11γ are involved in the regulation of peroxisome size and number (proliferation) in mammals. In *Y. lipolytica* (Pex23, Pex24) and *S. cerevisiae* (Pex25, Pex27-Pex32, Pex34, Pex35) several other peroxins have been identified which influence the size and number or organization of peroxisomes. Mammalian Pex11β remodels the peroxisomal membrane and interacts with the membrane adaptors Mff and Fis1, which recruit the dynamin-like fission GTPase Drp1 (DRP3A in plants, Vps1p, Dnm1p in *S. cerevisiae*) to peroxisomes, which is activated by Pex11β. Additional adaptor proteins are involved in yeast (Mdv1, Caf4) and plants (PMD1; see text). Motility and inheritance: mammalian peroxisomes move along microtubules, and Miro1 serves as membrane adaptor for the microtubule-dependent motor proteins kinesin and dynein. Inp1 and Inp2 are involved in the inheritance and motility of peroxisomes in *S. cerevisiae* and *Y. lipolytica*. Inp2 is the membrane receptor for the type V myosin motor Myo2 on peroxisomes, which drives peroxisomes along actin filaments. The GTPase Rho1 binds to Pex25 and is involved in the recruitment of actin to peroxisomes in *S. cerevisiae*. Tethering: ACBD5 and ACBD4 interact with ER-resident VAPA/B to mediate peroxisome–ER contacts in mammals. In yeast, Inp1, Pex3, Pex30 and Pex34 are involved in inter-organelle contacts (ER and mitochondria) (see also Fig. 3). Metabolite transport: uptake of fatty acids is mediated by ABC transporter proteins (ABCD1-3 in mammals; Pxa1-2 in yeast) (ALD, adrenoleukodystrophy protein; ALDR, ALD-related protein). Other transporter and membrane proteins/enzymes: OCTN3, organic cation/carnitine transporter 3; MCT1/2, monocarboxylate transporter 1/2; Opt2, yeast oligopeptide transporter (Elbaz-Alon et al. 2014); PMP52 (Tmem135) and PMP24 (PxmP4) belong to the Tim17 family (Žárský and Doležal 2016); members of the PMP22 family are Mpv17, Mpv17-like (ML-P), *S. cerevisiae* Sym1 (mitochondrial) and WSC (Woronin body sorting complex) in *N. crassa*; ACSL1/4, acyl-CoA synthetase long chain family member 1/4; Ant1, peroxisomal adenine nucleotide transporter 1; mARC2 (Mosc2), mitochondrial amidoxime reducing component 2; ATAD1/Msp1, ATPase family AAA (ATPase associated with various cellular activities) domain-containing protein 1; Atg37, autophagy-related protein 37 (Nazarko et al. 2014); FALDH, fatty aldehyde dehydrogenase (Costello et al. 2017a, b, c); FAR1, fatty acyl-CoA reductase 1 (ether lipid biosynthesis); GDAP1, ganglioside-induced differentiation-associated protein 1; MAVS, mitochondrial antiviral signalling protein; TRIM37, tripartite motif-containing protein 37; USP30, ubiquitin-specific protease 30 (Marcassa et al. 2018). Proteins with a dual localization to both peroxisomes and mitochondria are marked with an asterisk. Pex, peroxin; PMP, peroxisomal membrane protein

**Fig. 2:**
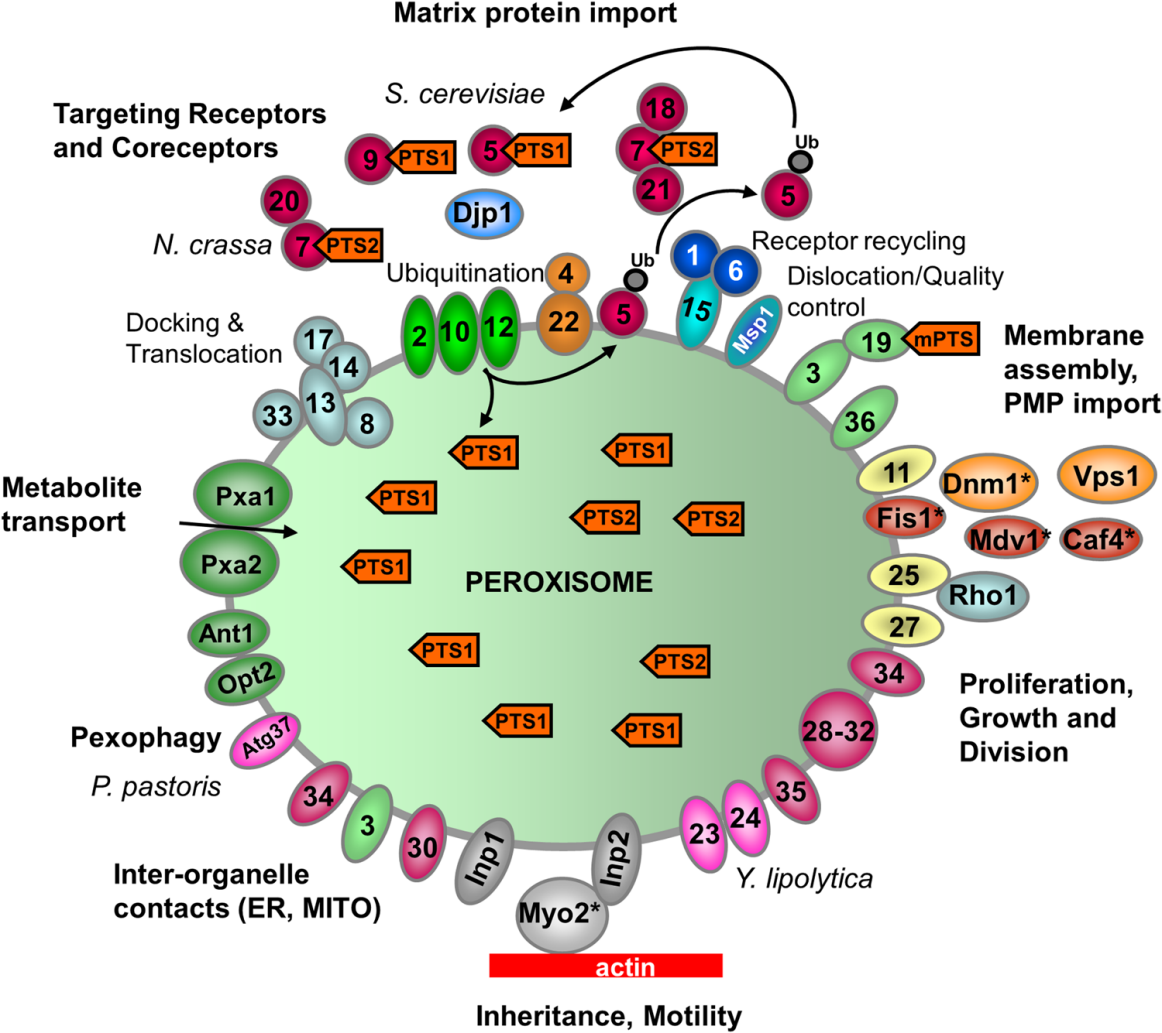
Schematic overview of the molecular machineries and proteins localized at the membranes of yeast peroxisomes. Adapted from (Schrader and Fahimi 2008). See legend Fig. 1 and text for further details

Peroxisome biogenesis involves the generation of the peroxisomal membrane and subsequent targeting and insertion of PMPs into the lipid bilayer, as well as the import of enzymes/proteins into the peroxisomal matrix. In contrast to other organelles such as mitochondria or the ER, peroxisomes can import completely folded and oligomeric or cofactor-bound proteins through a dynamic protein translocon (Meinecke et al. 2010; Montilla-Martinez et al. 2015; Dias et al. 2017). The import of matrix proteins and (most) PMPs involves largely conserved, but distinct import machineries with unique properties (Figs. 1, 2). Essential biogenesis factors, so-called peroxins (Pex proteins) form the import machineries. Since our last review in 2012, the number of identified peroxins has increased to 36. Pex9 is a new Pex5-like yeast peroxisomal targeting receptor for a subset of PTS1 (peroxisomal targeting signal)-containing matrix proteins during growth in oleate (Effelsberg et al. 2016; Yifrach et al. 2016). The existence of two distinct PTS1 receptors, Pex5 and Pex9 (in addition to PTS2-dependent import routes), allows yeast cells to adapt the metabolic capacity of peroxisomes to environmental changes. Pex35 was also identified as a new peroxisomal membrane protein in yeast, which is a regulator of peroxisome abundance (Yofe et al. 2017) whilst the new yeast peroxin, Pex36, a functional homolog of mammalian Pex16, functions in the ER-to-peroxisome traffic of PMPs (Farré et al. 2017). Finally, a role in the import of matrix proteins required for fatty acid β-oxidation and bile acid synthesis was proposed for the peroxisomal transmembrane protein TMEM135 (PMP52), which has high homology to the TIM17 family that mediates protein translocation across mitochondrial membranes (Renquist et al. 2018). TMEM135 was identified as a novel target of liver x receptors (LXRs), which belong to the nuclear receptor superfamily and are key regulators of cholesterol and fatty acid metabolism (Renquist et al. 2018).

With respect to matrix protein import, the processes of cargo translocation and receptor recycling are still debated (reviewed in Francisco et al. 2017). Progress has been made in the understanding of the unique structure and molecular function of the peroxisomal AAA–ATPase Pex1/Pex6 complex, which is involved in the export and recycling of the ubiquitinated import receptors Pex5 and Pex7 (Ciniawsky et al. 2015; Blok et al. 2015; reviewed in Schwerter et al. 2017) (Figs. 1, 2). Very recently, it was shown that the AAA–ATPase Pex1/Pex6 unfolds substrates by processive threading (Gardner et al. 2018), and that monoubiquitinated Pex5, which interacts with the AAA–ATPases Pex1 and Pex6, is unfolded during its dislocation to the cytosol (Pedrosa et al. 2018).

Interestingly, further evidence has now been provided that human Pex5 can function as a redox/stress sensor to retain peroxisomal catalase in the cytosol to combat oxidative stress of non-peroxisomal origin (Walton et al. 2017). Remarkably, small molecule inhibitors of peroxisomal (glycosomal) protein import (directed against Pex14) have been developed, which efficiently disrupt glycosomal matrix protein import in Trypanosoma parasites. This results in mislocalization of glycosomal enzymes, causing metabolic catastrophe and death of the parasite (Dawidowski et al. 2017). These are examples which link peroxisomal protein import to redox homeostasis and healthy ageing, and to the combat of parasites and the development of new therapies against trypanosomiases.

The import/insertion of PMPs depends on the membrane biogenesis factors Pex19, Pex16 and Pex3 (Fig. 1, 2). Excellent reviews on peroxisomal membrane biogenesis and PMP targeting and integration into the lipid bilayer have recently been published in a special issue on the assembly, maintenance and dynamics of peroxisomes published in *Biochim Biophys Acta*—Molecular Cell Research [Erdmann (Ed.) 2016]. This issue also contains comprehensive reviews about matrix protein import. New insight has meanwhile been obtained in the targeting, insertion and quality control of tail-anchored membrane proteins at peroxisomes. Recent studies support a direct, Pex19-dependent pathway (Yagita et al. 2013; Costello et al. 2017a, b, c) and a hydrophobic handoff mechanism for membrane insertion (Chen et al. 2014). Furthermore, targeting information in peroxisomal TA proteins has been revealed, and new peroxisomal TA proteins have been predicted and identified (Buentzel et al. 2015; Costello et al. 2017a, b, c). Those include the peroxisome–ER tether ACBD4 and the motor protein adaptor MIRO1 (see “Peroxisome–organelle interactions: the mysterious world of tethers” and “Peroxisome motility and distribution: mysterious movers”) (Fig. 1). Furthermore, a role for the AAA protein Msp1/ATAD1 in the clearance of excess tail-anchored proteins from the peroxisomal membrane has been revealed (Weir et al. 2017) (Fig. 1, 2). Many of those TA proteins, which act as membrane adaptors for important, disease-relevant cellular processes, are shared with mitochondria (see “Mysterious multiplication: new insights into peroxisome division” and “Peroxisome–organelle interactions: the mysterious world of tethers”) (Schrader et al. 2015a, b) (Fig. 1, 2).

### Peroxisome formation: mysterious with a new twist

It is now accepted that peroxisomes can form via the classical route of growth and division of pre-existing organelles, or via an alternate route of de novo formation of nascent peroxisomes (for recent reviews see Hettema et al. 2014; Agrawal and Subramani 2016). The latter pathway is based on studies in mutant cells lacking peroxisomes due to a loss of the membrane biogenesis factors Pex3, Pex16 or Pex19. However, peroxisome numbers appear to be primarily controlled by growth and division (Motley and Hettema 2007). The de novo model suggests that several key PMPs (e.g. Pex3) target the ER, sequester into pre-peroxisomal vesicles, which are released and form import-competent peroxisomes which then grow and divide to multiply (Hoepfner et al. 2005). There was some debate about the initiation of de novo formation at the ER, as pre-peroxisomal vesicles were also observed in yeast cells lacking Pex3 or Pex19. These vesicles were degraded by autophagy and had, therefore, been overlooked (Knoops et al. 2014; Wróblewska et al. 2017). Recent studies in yeast have, however, revealed a role for the reticulon-like proteins Pex30 and Pex31 in the generation of an ER subdomain in which pre-peroxisomal vesicles bud (David et al. 2013; Mast et al. 2016; Joshi et al. 2016). Furthermore, a role for ESCRT-III proteins Vps20 and Snf7 in the release of pre-peroxisomal vesicles from the ER was identified (Mast et al. 2018), supporting the ER origin of pre-peroxisomal vesicles. In addition, Pex36, a new yeast peroxin and functional homolog of mammalian Pex16, has been identified, which functions in ER-to-peroxisome trafficking of peroxisomal membrane proteins (Farré et al. 2017) (Fig. 1, 2).

**Fig. 3:**
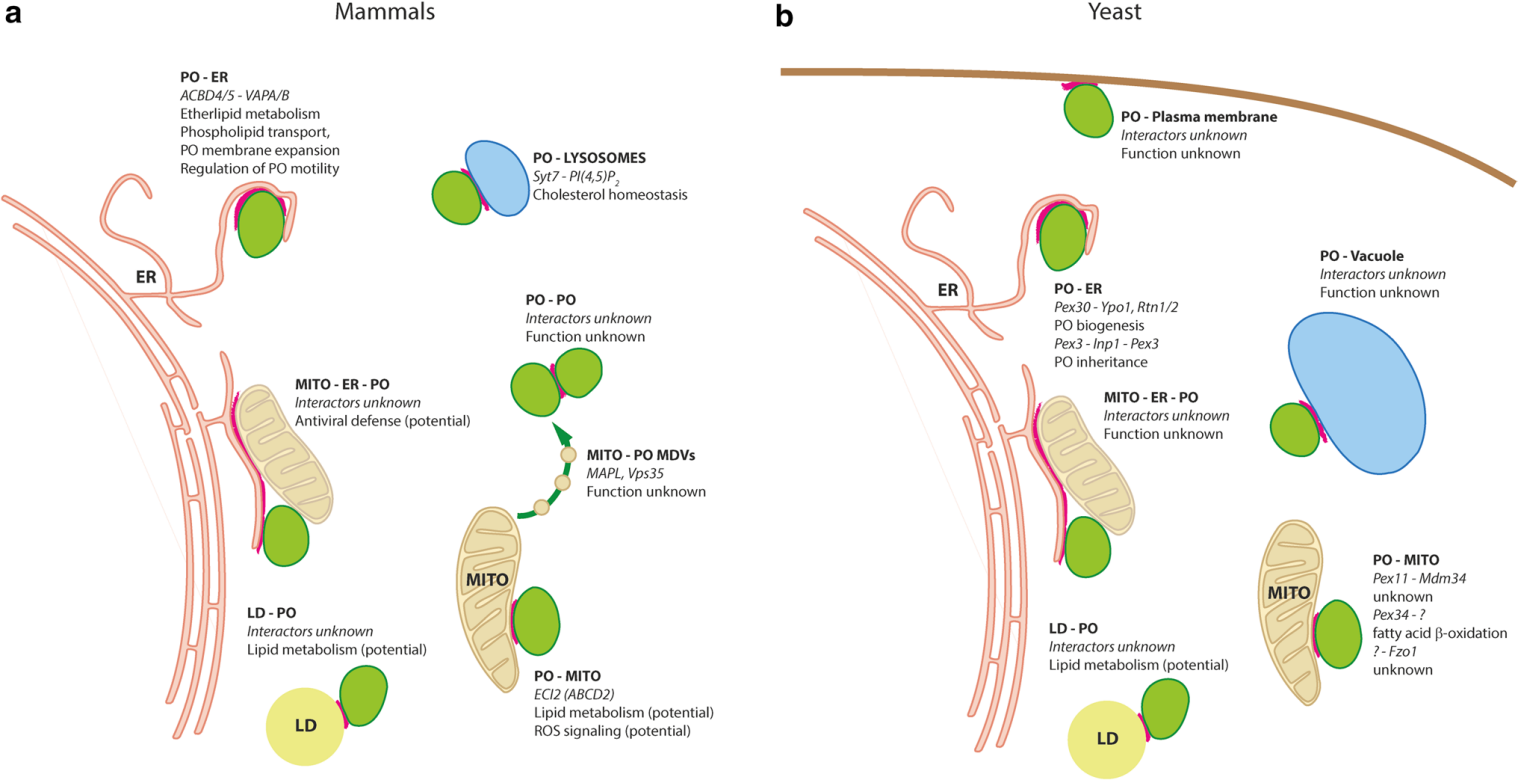
Contact zones between peroxisomes and other organelles described in mammals and yeast species. Identified tethering complexes and (hypothetical) associated functions are shown next to the symbolized interactions. **a** In mammalian species, peroxisome interactions have been reported for the ER (Costello et al. 2017; Hua et al. 2017), mitochondria (Neuspiel et al. 2008; Braschi et al. 2010; Fan et al. 2016), lysosomes (Chu et al. 2015), lipid droplets (Schrader 2001; Valm et al. 2017), peroxisomes themselves (Bonekamp et al. 2012) and the ER + mitochondria in triple contacts (Horner et al. 2015). **b** For yeasts, peroxisome interactions have been described for the plasma membrane (Shai et al. 2018), the ER (Knoblach et al. 2013; Mast et al. 2016), mitochondria (Mattiazzi Ušaj et al. 2015; Shai et al. 2018), the vacuole (Shai et al. 2018), lipid droplets (Binns et al. 2006) and ER + mitochondria (Cohen et al. 2014). PO, peroxisomes; MITO, mitochondria; LD, lipid droplets

Studies with human patient fibroblasts lacking Pex3 or Pex16, which are devoid of peroxisomes, added another twist to the model of de novo biogenesis (Sugiura et al. 2017). When Pex3 was re-introduced, it targeted mitochondria and was released in pre-peroxisomal vesicles. Pex16, on the other hand, targeted the ER, where it exited in pre-peroxisomal vesicles that appeared to fuse with the mitochondria-derived pre-peroxisomes to generate new, import-competent peroxisomes (Sugiura et al. 2017). Thus, both ER and mitochondria can contribute to the de novo formation of peroxisomes in mammalian cells. The initial targeting of PMPs in the absence of peroxisomes may, therefore, be a key event in de novo formation (for recent reviews/commentaries, see Hettema and Gould 2017; Schrader and Pellegrini 2017; Costello and Schrader 2018). The ER-derived biogenic route and the physiological role of the de novo pathway are still controversially discussed, but it is recognized that the ER makes important contributions to peroxisome biogenesis and that peroxisomes are semi-autonomous organelles, which depend on other cellular compartments such as the ER to obtain lipids or even specific proteins (see “Peroxisome–organelle interactions: the mysterious world of tethers”) (Titorenko and Rachubinski 2014) (Fig. 3).

### Mysterious multiplication: new insights into peroxisome division

Peroxisomes are dynamic organelles which can multiply by membrane growth and division of pre-existing organelles (reviewed in Islinger et al. 2012a, b; Schrader et al. 2016). This involves remodelling and expansion of the peroxisomal membrane through the formation of tubular membrane extensions which then constrict and divide into new peroxisomes. In mammals, this is supposed to be an asymmetric process, which forms new peroxisomes via generation of a membrane compartment and subsequent import of newly synthesized matrix proteins (Huybrechts et al. 2009; Delille et al. 2010). The membrane peroxin Pex11β is a key factor in the regulation of peroxisome number in mammals, which has now been associated with all steps of peroxisomal growth and division (Fig. 1, 2). Through oligomerisation and interaction with membrane lipids via N-terminal amphipathic helices, Pex11β acts as a membrane-shaping protein which remodels, deforms and elongates the peroxisomal membrane prior to fission (Opaliński et al. 2011; Yoshida et al. 2015, Su et al. 2018). Pex11β also interacts with the membrane adaptors Fis1 (fission protein 1) and Mff (mitochondrial fission factor) at the peroxisomal membrane, which recruit the dynamin-related fission GTPase Drp1, thus contributing to the assembly of the peroxisomal division machinery (reviewed in Koch and Brocard 2012; Itoyama et al. 2013; Schrader et al. 2016) (Fig. 1, 2). Furthermore, it has been revealed that Pex11β functions as a GTPase-activating protein (GAP) for Drp1 during peroxisomal fission (Williams et al. 2015). How peroxisomal membranes constrict prior to final membrane scission by Drp1 is still unclear; however, it is possible that Pex11β is also involved in constriction. For mitochondria, a role of the ER in membrane division has been revealed (Friedman et al. 2011; Lewis et al. 2016). If the same applies to peroxisomes is currently unknown. Our knowledge about key proteins in peroxisome division and multiplication has clearly increased, but it will be a challenge for upcoming years to understand their coordinated interplay and regulation.

An important discovery in the field was that peroxisomes and mitochondria share proteins of their division machinery, for example Fis1 (Koch et al. 2005), Mff (Gandre-Babbe and van der Bliek 2008), the ganglioside-induced differentiation-associated protein GDAP1 (Huber et al. 2013), and Drp1 (Li and Gould 2003; Koch et al. 2003) in mammals (Fig. 1). Sharing of division factors between peroxisomes and mitochondria has also been reported in other organisms, e.g. for the plant-specific division factor PMD1 (peroxisomal and mitochondrial division factor 1) (Aung and Hu 2011), and for the adaptors Mdv1 and Caf4 as well as the dynamin-related GTPase Dnm1 in the yeast *S. cerevisiae* (Kuravi et al. 2006; Motley et al. 2008) (Fig. 2). PMD1 has very recently been reported to influence peroxisome proliferation upon salt stress in *Arabidopsis thaliana* (Frick and Strader 2018). For reviews on peroxisome division and proliferation in plants and yeast, see Hu (2010) and Saraya et al. (2010). Sharing division components between peroxisomes and mitochondria is seen as a common, evolutionarily conserved strategy amongst mammals, fungi and plants, contributing to the “peroxisome–mitochondria connection”, which impacts on their cooperative functions and contribution to diseases, and promotes healthy lifespan (Waterham et al. 2007; Shamseldin et al. 2012; Schrader et al. 2015a, b; Koch et al. 2016; Weir et al. 2017a, b).

Meanwhile, several patients with defects in the peroxisomal division/dynamic proteins Drp1, Mff and Pex11β have been identified (reviewed in Costello et al. 2018). Drp1 and Mff deficiencies usually impair both peroxisomal and mitochondrial division resulting in highly elongated organelles. Drp1 deficiency, the first disorder described with a defect in both mitochondrial and peroxisomal fission (Waterham et al. 2007), combined clinical features of peroxisomal (dysmyelination, severity) and mitochondrial disorders (autosomal dominant optic atrophy, neuropathy). Genetic analysis of this first patient, who died only a few weeks after birth, revealed a heterozygous, dominant-negative missense mutation (Ala395Asp) in the middle domain of Drp1, which inhibits Drp1 oligomerization and subsequent function in membrane fission (Chang et al. 2010). Additional Drp1 patients, who presented with developmental delay, refractory epilepsy or infantile encephalopathy, were recently described (Yoon et al. 2016; Chao et al. 2016; Sheffer et al. 2016; Vanstone et al. 2016; Fahrner et al. 2016; Nasca et al. 2016; Zaha et al. 2016). Genetic analysis revealed (1) missense variants in the Drp1 middle (oligomerisation) domain (Gly362Asp, G350R, E379K) implying a dominant-negative mechanism, (2) recessive nonsense mutations leading to truncated unstable protein (Chao et al. 2016; Sheffer et al. 2016; Vanstone et al. 2016), or (3) the first dominantly inherited mutations in Drp1 affecting conserved amino acids within the Drp1 GTPase domain (Gerber et al. 2017). The latter Drp1 missense mutations were linked to the blinding disease optic atrophy. However, whereas mitochondria were elongated in patient fibroblasts, peroxisome morphology appeared normal (Gerber et al. 2017). The first patients with Mff deficiency due to loss-of-function mutations in the Mff gene were reported (Shamseldin et al. 2012; Koch et al. 2016). They presented with developmental delay, peripheral neuropathy, optic atrophy, and Leigh-like encephalopathy. Mitochondria and peroxisomes are highly elongated in patient fibroblasts, due to a failure in organelle division. Of note, Mff was also identified as a key effector of energy-sensing adenosine monophosphate (AMP)-activated protein kinase (AMPK)-mediated mitochondrial fission (Toyama et al. 2016). In contrast to the neurological features observed in Mff patients, Mff-deficient mice die as a result of severe dilated cardiomyopathy leading to heart failure, which is likely the result of mitochondrial defects (Chen et al. 2015). Whereas mitochondria and peroxisomes in Mff-deficient mouse embryonic fibroblasts were highly elongated, their length was not substantially altered in Mff-deficient mouse cardiomyocytes. However, an increased heterogeneity in mitochondrial shape and abundance was observed (Chen et al. 2015). This may indicate that peroxisomal (mitochondrial) morphology and division is affected in a cell type-specific manner. A mathematical modelling approach was recently developed to explain and predict alterations in peroxisome morphology and dynamics in health and disease conditions (Castro et al. 2018a, b).

Patients with a loss of Pex11β present with short stature, eye problems (congenital cataracts), progressive hearing loss and neurological defects (Ebberink et al. 2012; Taylor et al. 2017). Peroxisome number and morphology in patient fibroblasts are altered. However, similar to Drp1 and Mff deficiency, the metabolic functions of peroxisomes are not significantly affected. This is in contrast to the classical peroxisome biogenesis disorders (e.g. Zellweger syndrome) and can complicate diagnosis through metabolic biomarkers (e.g. VLCFA). It also suggests that the patients’ symptoms relate to defects in peroxisome dynamics and plasticity, highlighting the importance of proper control of peroxisome abundance and membrane dynamics for cellular function. Interestingly, altered peroxisome abundance in Pex11β-deficient epidermal cells was recently reported to result in abnormal mitosis and organelle inheritance, thus affecting cell fate decisions (Asare et al. 2017).

### Peroxisome motility and distribution: mysterious movers

Progress has also been made in the understanding of peroxisome motility and the role of the cytoskeleton in peroxisome dynamics and distribution. In baker’s yeast, peroxisomes move along actin filaments by recruiting the myosin V motor Myo2 via the PMP Inp2 (Inheritance protein 2) (Fig. 2). This is crucial for the transport of peroxisomes into the bud, and thus for peroxisome inheritance. For balanced distribution, Inp1, another inheritance protein, links peroxisomes to the peripheral ER, thus retaining some peroxisomes in the mother cell (reviewed in Knoblach and Rachubinski 2015, 2016). Plant cells also move peroxisomes via actin filaments and myosin motors (reviewed in Sparkes and Gao 2014). PMD1 which is required for NaCl-induced peroxisome division (see above) is also an actin-binding protein and may mediate the peroxisome–cytoskeleton connection in plants (Frick and Strader 2018). In contrast, in mammalian cells peroxisomes move bidirectionally along microtubules, using both kinesin and dynein motors (reviewed in Schrader et al. 2003; Neuhaus et al. 2016). How microtubule motors are recruited to peroxisomes in mammalian cells was long unclear, but recently a role for the mitochondrial Rho GTPase Miro1 was revealed (Castro et al. 2018a, b; Okumoto et al. 2018) (Fig. 1). Miro1, which was initially described as a mitochondrial membrane adaptor for kinesin, is also targeted to peroxisomes, contributing to peroxisome distribution and microtubule-dependent motility. Like Fis1, Mff, GDAP1, MAVS and *At*PMD1, Miro1 is also a tail-anchored membrane adaptor, which is shared by mitochondria and peroxisomes (Costello et al. 2017a, b, c) (Fig. 1). Interestingly, Miro1-mediated pulling forces also contribute to peroxisome membrane elongation and proliferation in cellular models of peroxisome disease (Castro et al. 2018a, b). These observations in combination with a mathematical model of peroxisome dynamics now allow us to link the microtubule cytoskeleton and motor-mediated pulling forces to peroxisome formation by growth and division in mammalian cells (Castro et al. 2018a, b). As peroxisome elongation and division can still occur in the absence of microtubules, this link was controversial. However, it is suggested that independent, but cooperative mechanisms exist, and that motor forces support membrane dynamics by providing directionality. This is now in agreement with observations in yeast, where actin-based myosin-driven pulling forces cause peroxisome elongation and separation in dynamin mutants (Hoepfner et al. 2001; Nagotu et al. 2008). In line with these observations, it is also possible that mechanical forces can divide peroxisomes, as this was recently reported for mitochondria (Helle et al. 2017). In this scenario, Mff is suggested to act as a membrane-bound force sensor to recruit the fission machinery to mechanically strained mitochondrial sites.

Similar to mammalian cells, peroxisomes in filamentous fungi also move along microtubules (reviewed in Knoblach and Rachubinski 2016; Steinberg 2016; Salogiannis and Reck-Peterson 2017). However, instead of binding motor proteins directly, they interact with motile early endosomes (EEs) (Guimaraes et al. 2015). In *A. nidulans* this “hitchhiking” on EEs requires PxdA, an EE-bound linker protein, which mediates peroxisome–EE interaction (Salogiannis et al. 2016). In the corn smut fungus *Ustilago maydis*, constant EE motility also enhances the diffusive motions of peroxisomes, which is supposed to impact on local mixing and organelle–organelle interactions (Lin et al. 2016). Mathematical modelling of various aspects of intracellular transport in the filamentous fungus *U. maydis* has revealed new insights into the spatial organization of peroxisomes and other organelles (Lin et al. 2016; Lin and Steinberg 2017). It showed that peroxisome mobility and mixing requires both, active diffusion and directed transport. These mechanisms ensure even distribution of peroxisomes and allow frequent interaction, which is important for proper cellular function.

### Peroxisome–organelle interactions: the mysterious world of tethers

An emerging theme in cell biology is the cooperation, communication and interaction between subcellular organelles, which often involve physical contacts via membrane contact sites. Peroxisomes are not isolated entities in the cell but communicate and share signals, metabolites and proteins with other compartments including the ER, mitochondria, lipid droplets and lysosomes (for recent reviews see Schrader et al. 2015; Wanders et al. 2016; Shai et al. 2016; Yoboue et al. 2018; Castro et al. 2018) (Fig. 3). Investigating organelle interaction and identifying proteins that mediate organelle contacts is an active research area, which requires novel tools and techniques. For example, the use of multi-spectral imaging allowed the simultaneous visualization of six organelles (including peroxisomes) and mapping of their interaction (Valm et al. 2017). Systematic mapping of contact sites in baker’s yeast by a proximity detection method based on split fluorophores revealed new contacts between peroxisomes and the plasma membrane, as well as between peroxisomes and the vacuole (Shai et al. 2018) (Fig. 3). Furthermore, individual tethering functions for the yeast mitofusin Fzo1 and the PMP Pex34 in peroxisome–mitochondria contacts were revealed (Fig. 3). This study also demonstrated a physiological role for peroxisome–mitochondria contacts in the β-oxidation of fatty acids, a process that requires metabolic cooperation between both organelles (for recent reviews on the peroxisome–mitochondria connection see Schrader et al. 2015a, b; Wanders et al. 2016; Pascual-Ahuir et al. 2017; Fransen et al. 2017). In this context it is of interest that in *S. cerevisiae* peroxisomes can be localized adjacent to a specific mitochondrial niche near the ER–mitochondria contact site, proximal to where the pyruvate dehydrogenase complex is found in the mitochondrial matrix, thus suggesting a three-way organelle junction. Peroxisomal Pex11 and mitochondrial Mdm34, one of the proteins creating the ER–mitochondria tether (ERMES), are supposed to mediate the peroxisome–mitochondria contact (Cohen et al. 2014) (Fig. 3). Comparable joined three-way organelle complexes between peroxisomes, chloroplasts and mitochondria have been observed in green cotyledon cells of *A. thaliana* upon exposure to light (Hayashi et al. 2001). Pex10 and an unknown counterpart on the chloroplast are supposed as mediators of the interaction, which overtly serve to shuttle photosynthesis products through the photorespiratory pathway during photorespiration. To date, direct contacts between peroxisomes and mitochondria in mammalian cells are not documented without any doubt. Yet, hormone-induced, controlled steroid hormone biosynthesis requires inter-organelle cooperation between peroxisomes and mitochondria. Using immunofluorescent staining and live-cell imaging, evidence was provided that di-butyryl-cAMP treatment of MA-10 mouse tumor Leydig cells rapidly induces peroxisomes to approach mitochondria (Fan et al. 2016). The authors suggest that isoform A of the endogenous acyl-CoA binding protein ACBD2/ECI2, head to tail inserted into peroxisomes and mitochondria, may play a role in establishing a two-way communication between both organelles for supplying cholesterol used for steroid hormone biosynthesis.

Machine learning prediction approaches in combination with mutational analyses revealed new tail-anchored adaptor proteins at peroxisomes and other organelles (Costello et al. 2017a, b, c) (Fig. 1). This then led to the molecular characterisation of the first peroxisome–ER membrane contact sites in mammalian cells involving the peroxisomal acyl-CoA-binding domain proteins ACBD5 and ACBD4, which interact via FFAT-like (two phenylalanines (FF) in an acidic tract) domains with ER-resident VAPA/B proteins, which are also tail-anchored membrane adaptors (Costello et al. 2017b, c; Hua et al. 2017) (Figs. 1, 3). The ACBD5–VAPA/B contact plays a role in plasmalogen biosynthesis, which requires metabolic cooperation between peroxisomes and the ER (Hua et al. 2017; Herzog et al. 2017). Moreover, the peroxisome–ER contacts influence peroxisome motility providing a new role for a peroxisome–ER tether in the regulation of peroxisome movement and membrane dynamics in mammalian cells (Costello et al. 2017; Hua et al. 2017; Castro et al. 2018a, b) (Fig. 3). In addition, a role of peroxisome–ER contacts in lipid transfer for peroxisome membrane expansion and biogenesis was revealed (Fig. 3). As discussed above, expansion and growth of the peroxisomal membrane is a prerequisite for division and proliferation. This requires lipids which are supposed to be provided by the ER in a non-vesicular pathway (Raychaudhuri and Prinz 2008; Costello et al. 2017). Defects in peroxisome division (e.g. due to loss of Mff or Drp1 function) result in highly elongated peroxisomes, suggesting a constant transfer of lipids from the ER to peroxisomes. Loss of peroxisome–ER interactions was shown to reduce membrane expansion supporting a role of peroxisome–ER contacts in lipid transfer for peroxisome biogenesis. To reveal how lipids are transferred is a challenging task for future studies. As approx. 70–80% of peroxisomes in cultured mammalian cells interact with the ER, ER-derived pre-peroxisomal vesicles may not have a major role in lipid transport to peroxisomes.

As a result of these studies, patients with mutations in ACBD5 have been identified, which suffer from retinal dystrophy and white matter disease (Yagita et al. 2017; Ferdinandusse et al. 2017); mutations in VAPB have been linked to amyotrophic lateral sclerosis (Taylor et al. 2016). This may suggest possible links between loss of peroxisome contact sites and cell dysfunction (reviewed in Castro et al. 2018).

Unveiling a hitherto undescribed organellar cooperation, `tethers`, shared by peroxisomes and the lysosomal compartments mediating the intracellular routing of cholesterol have been described (Du et al. 2015). Cholesterol is an essential determinant of membrane fluidity, permeability and organization in animal cells (Chang et al. 2006). With the vast majority localized in the plasma membrane (Maxfield and Wüstner 2002), it originates from the ER via de novo synthesis (Horton et al. 2002; Kovacs et al. 2002), and from lysosomes via receptor-mediated endocytosis of plasma LDL (Brown and Goldstein 1986). It plays important roles in steroidogenesis, bile acid biosynthesis and signal transduction by regulatory oxysterols (Yeagle 1988; Lingwood and Simons 2010), implying a dynamic intracellular routing. This raises the fundamental question, `how is cholesterol transported from compartment to compartment`? Surprisingly, peroxisomes have been shown to play a critical part in the transport of free cholesterol from lysosomes to the plasma membrane (Chu et al. 2015). A so-far unrecognized contact between lysosomes and peroxisomes was observed, established at least in part by the binding of the integral lysosomal membrane protein synaptotagmin 7 to the lipid PI(4,5)P_2_ on the peroxisomal membrane (Fig. 3). Notably, efficient formation of the tether required NPC1, which proved to be transient and cholesterol dependent. Disruption of critical peroxisomal genes led to an accumulation of cholesterol in lysosomes as it is observed in the Niemann–Pick disease type C (NPC). The latter is a fatal predominantly neurodegenerative disorder (Carstea et al. 1997) caused by mutations in NPC1 and NPC2, which together mediate the transport of free cholesterol out of the lumen to the limiting membrane of lysosomes (Sleat et al. 2004). How cholesterol finally approaches the plasma membrane is still elusive. In any case, peroxisomes have apparently a pivotal role in the intracellular trafficking of cholesterol and its derivatives.

Dietary uptake, endogenous de novo synthesis, efflux and conversion of cholesterol to derivatives like bile acids, tightly regulate cellular cholesterol levels. An elaborate feedback system senses the actual concentration adjusting it by both trans- as well as post-transcriptional systems. Central to the transcriptional control are (1) the sterol regulating element-binding protein (SREBP) family; (2) the SREBP cleavage-activating protein (SCAP) which functions as a sterol sensor; (3) the Insigs (insulin-induced genes) which control SREBPs over a wide range of cholesterol concentrations. The Insig–SCAP–SREBP network resides in the ER with its very low levels of sterols. In studies using a mouse model for Zellweger syndrome (Pex2^−/−^ mice), low levels of cholesterol in plasma and liver of the mice were observed. Moreover, the mice were unable to maintain normal cholesterol homeostasis despite activation of the master regulator SREBP and increased activities of cholesterol biosynthetic enzymes. Last but not least, the SREBP complex remained activated even after normalization of hepatic cholesterol in response to bile acid feeding (Faust and Kovacs 2014). In line with preceding studies (Kovacs et al. 2009), the authors suggest that peroxisome deficiency activates hepatic ER-stress pathways leading to a dysregulation of the endogenous sterol-response mechanism.

## Mysterious messengers: peroxisomes in signalling and antiviral defence

Importantly, peroxisomes cooperate in antiviral signalling and defence by means of the tail-anchored MAVS (mitochondrial antiviral signalling) proteins (Dixit et al. 2010; Kagan 2012) (Fig. 1). Thereby, peroxisomal MAVS rapidly induce expression of a subset of antiviral genes that curb viral replication, while the mitochondrial MAVS induce a sustained antiviral response. Interestingly, peroxisomes are also required for the engulfment of bacteria by *Drosophila* and mouse macrophages, and the resolution of bacterial infections by modulating the canonical innate immunity pathways through ROS and RNS signalling (Di Cara et al. 2017). The role of peroxisomes was investigated in adult *Drosophila* flies and the related S2 cell line as well as in mice, in which consistently the key peroxins Pex5 and Pex7 had been impaired. A reduced capacity in responding to microbial pathogens, defects in immune signalling, and a reduced viability have been observed in flies and S2 cells, and a requirement for peroxisomes in microbe engulfment by the murine macrophages could be documented. Interpreting the findings related to the impaired expression of the peroxins, the authors suppose a compromised phagocytosis of bacteria, defects in the reorganization of the cytoskeleton required for forming phagosomes and proceeding phagocytosis, and a modulation in ROS/RNS signalling to activate an immune response. It is tempting in line with the forgoing reports (e.g. Dixit et al. 2010) to cede peroxisomes a critical subcellular hub in promoting innate immune responses.

HIV viruses are particularly successful in subverting host antiviral responses. It has recently become apparent that peroxisomes are part of these effective countermeasures (Xu et al. 2017). Specifically, HIV-infected cells express high levels of microRNAs, a subset of which are predicted to target peroxisome biogenesis factors (PEX2, PEX7, PEX11ß, PEX13) resulting in reduced numbers of peroxisomes. Interestingly, levels of these microRNAs proved to be elevated in brain tissues from HIV patients as well as HIV-infected macrophages. Thus, increasing the expression of microRNAs that down-regulate peroxisomes might be a novel mechanism to interfere with early antiviral signalling emanating from these organelles. The development of neurological disorders in AIDS patients might be attributed to this mechanism.

Reactive oxygen/nitrogen species (ROS/NOS) in concert with other reactive molecules have emerged over the past decades as important regulators of many physiological and pathological processes, contributing to and completing superior regulating systems operating in a living organism. ROS/NOS serve as signalling messengers, mediating various biological responses including gene expression, cell proliferation, angiogenesis, innate immunity, programmed cell death and senescence (Dowling and Simmons 2009; Scherz-Shouval and Elazar 2011). On the other hand, increased levels of these short-lived reactive molecules or any disturbance in ROS/NOS homeostasis can exert harmful effects due to oxidative stress (e.g. Salmon et al. 2010).

In the past years, peroxisomes have been pointed out as key regulators in overall cellular lipid and ROS/NOS metabolism, thereby intimately interacting both functionally and physically with other cell organelles, in particular with mitochondria (Fransen et al. 2017). Plant peroxisomes have been shown to house principal enzymes involved in the generation of ROS/NOS, related to the defence against oxidative stress (Corpas et al. 2017). Seed germination in the dark requires enzymes catalysing β-oxidation and gluconeogenesis to convert fatty acids into sugars, and the components of the ascorbate–glutathione cycle to protect oil bodies against oxidative damage caused by H_2_O_2_ produced during the breakdown of the fatty acids (Eastmond 2007; Goepfert and Poirier 2007). In leaves, stomatal movement is highly regulated by external stimuli (light) as well as internal molecules (hormones). NO induces stomatal closure, and restricts the entry of pathogenic microorganisms (Neill et al. 2008). Peroxisomal NO and ROS are involved in leaf senescence, characterized by a decrease in catalase activity and a down-regulation of NO generation (Corpas et al. 2004). In *A. thaliana* seedlings grown under salinity stress or exposed to cadmium, an increase in peroxisomal NO content has been reported (Corpas and Barroso 2014). In summary, these findings highlight the importance of peroxisomal NO metabolism under abiotic stress conditions in plants. ROS production in plant cells shows dramatic increases during senescence and under biotic and abiotic stress (Zentgraf 2007). Within this scenario, H_2_O_2_ plays a key role. Ascorbate peroxidase is probably the most important enzyme scavenging H_2_O_2_ produced in chloroplasts, yet is also present in cytoplasm, peroxisomes and mitochondria (Narendra et al. 2006). In *Arabidopsis* different catalase isoforms are described with Cat3 levels varying substantially during the plant life span, increasing particularly in leaves of senescent plants (Zimmermann et al. 2006). Matching these variations, it could be demonstrated by in vivo imaging that peroxisomal H_2_O_2_ in leaves is also modulated during the life cycle (Costa et al. 2010). Interestingly, clear evidence could be provided for a strict correlation between Cat3 expression levels and effective H_2_O_2_ scavenging dependent on intra-peroxisomal Ca^2+^. Apparently, activation of Cat3 caused by an increase of Ca^2+^ inside peroxisomes represents a highly efficient cellular mechanism to strictly control H_2_O_2_ levels.

It is widely accepted that an accumulation of senescent cells and accompanying secretions as well as the loss of stem cell renewal capacities contribute to tissue ageing (Collado et al. 2007). The existence of these cells in tissues of ageing primates was confirmed about a decade ago (Herbig et al. 2006), and their elimination in a mouse model indeed delayed the appearance of age-related disorders (Baker et al. 2011). Senescent cells have lost their ability to replicate, are enlarged, and express so-called senescence markers. Moreover, they are resistant to apoptosis and secrete bioactive molecules, e.g. cytokines (Giordano and Terlecky 2012). Ageing is considered a natural phenomenon in which cells enter into a senescent stage to avoid transformation into cancerous cells (Campisi and Robert 2014). Multiple factors affect cellular ageing including shortening of telomeres, alteration of protein expression, defects in DNA repair machinery and accumulation of cellular ROS—in particular H_2_O_2_—which are suggested a “primary mediator” of in vitro senescence and in vivo ageing (Lu and Finkel 2008). In view of their diverse metabolic functions, in particular their role both as source and sink of ROS in a cell, peroxisomes are a predestined hub in cellular ageing. Indeed, a plethora of studies employing cell lines of diverse origin, animals, and yeast cultures report on profound alterations in the biogenesis and proliferation of the organelle, in the rate of expression as well as location of peroxisomal enzymes engaged in ROS metabolism (catalase), and last but not least in the interaction with other cell organelles, particularly mitochondria (Deori et al. 2018 and ref. therein). Consistently, they reveal that peroxisomes are critical contributors to ageing, longevity and age-related disorders.

The free radical theory of ageing posits oxidative damage to macromolecules as a primary determinant of lifespan. In some cases, however, longevity is enhanced by the inactivation of oxidative stress defences or is correlated with increased, rather than decreased ROS and oxidative damage. Using *S. cerevisiae*, Mesquita et al. convincingly demonstrated that caloric restriction or inactivation of catalase induces oxidative stress by H_2_O_2_, nevertheless promoting longevity despite increased oxidative damage of macromolecules (Mesquita et al. 2010). An induction of superoxide dismutase by H_2_O_2_ reducing the levels of oxygen radicals was proposed to account for this surprising finding pointing to a hormesis effect of H_2_O_2_ in promoting longevity.

Another regulatory effect of peroxisomal ROS with a profound impact on cellular growth was reported some years ago (Zhang et al. 2013). Two components (TSC1 and TSC2) of the tuberous sclerosis complex (TSC) were found to localize to peroxisomes. In response to ROS both influence mTorC1, which upon diverse inputs (insulin, glucose, amino acids) affects the switch between growth and autophagy. According to these observations, peroxisomes have an impact on the central regulator of cellular growth in mammalian tissues.

Type 2 diabetes is a complex disease accompanied by elevated levels of non-esterified fatty acids (NEFAs). The latter are known to disturb the function of β-cells and to induce loss of these cells, effects termed lipotoxicity. In a study employing primary rat islet cells as well as related cell lines, experimental evidence has been provided that NEFA-induced β-cell lipotoxicity is intimately related to peroxisomal metabolism of NEFAs (Elsner et al. 2011). Since the expression of H_2_O_2_-inactivating catalase is virtually absent in peroxisomes of insulin-secreting β-cells (Lenzen et al. 1996), the inactivation of H_2_O_2_ generated in peroxisomes by the β-oxidation of NEFAs is severely impeded, explaining the exceptional susceptibility of pancreatic β-cells to lipotoxicity.

Summarizing the findings on the functional plasticity of peroxisomes, we are overtly stepping from the view of a relict “fossil organelle” towards an extremely important one for optimum functioning of a cell. Despite great advances in unravelling its diverse contributions to the vitality-respective abiosis of cells, it is evident that peroxisomes will continue to emerge as critical contributors to these fundamental features.

## News from the brain: unravelling the mysterious role of peroxisomes in the central nervous system (CNS)

One of the major hallmarks of peroxisomal inherited disorders is the often severe neuropathological phenotype represented by developmental alterations in neuronal migration, a progressive demyelination of neurons or inflammatory activation of microglia (Berger et al. 2016). The significance of peroxisomal metabolism for the maintenance of brain physiology is evident, but several open questions remain: (1) peroxisomes in different neural cell types and brain regions show heterogeneity (Ahlemeyer et al. 2007) pointing to different functions in the individual cell types which may contribute differently to the phenotype of peroxisomal disorders. (2) Differences in brain pathology of peroxisomal biogenesis disorders (PBDs) and the single enzyme deficiencies (SEDs) suggest that not only a single metabolic function of peroxisomes is responsible for disease pathogenesis. It remains to be clarified how the distinct metabolic pathways of peroxisomes and/or regulatory functions contribute to the development and maintenance of the CNS. (3) Although the aetiology of various inherited peroxisomal disorders has been clarified, there is still significant lack of information on the disease mechanism and active metabolites disturbing cellular physiology. (4) While alterations in mitochondria and the ER have been associated with the pathogenesis of important neurodegenerative diseases such as Huntington’s, Parkinson’s and Alzheimer’s disease (Xiang et al. 2017; Martinez-Vicente 2017), a possible contribution of peroxisomes to the pathology of these diseases is largely unexplored. Since our last review several of these open questions have been addressed in a variety of studies summarized below.

To evaluate the role of peroxisomes for maintaining brain pathology, the groups of Nave and Baes created several conditional mouse knockout strains deleting the peroxisomal import receptor Pex5 from the liver, all neural cell types, oligodendrocytes, astrocytes and projection neurons, respectively (Krysko et al. 2007; Kassmann et al. 2007; Bottelbergs et al. 2010). While disruption of peroxisomal functions in liver resulted in the most severe phenotype, showing developmental changes in brain architecture, deletion of PEX5 from all neural cells exhibited only a developmental delay in neural cell migration and primarily induced degenerative alterations in axons in adulthood (Krysko et al. 2007; Hulshagen et al. 2008). Comparably, brain-specific conditional Pex13^−/−^ mice exhibit a developmental phenotype with delayed formation of cerebellar layers but additionally showed abnormal Purkinje cell differentiation accompanied by a reactive gliosis (Müller et al. 2011). At later stages, these knockout mice showed a degeneration of serotonergic neurons in Raphe nuclei (Rahim et al. 2014). The neurons exhibited abnormal axonal swellings which were accompanied by an activation of astro- and microglia indicating inflammatory processes. In addition to functions in the CNS locomotor system, serotonergic Raphe neurons contribute to neuro-vegetative control and emotional behaviour (Lucki 1998), brain functions which have hitherto not been investigated in the light of peroxisomal disorders.

With regard to the importance of peroxisomal metabolism in individual neural cell types, specific deletion of peroxisomal function in oligodendrocytes appeared to be most crucial for maintaining brain homeostasis. In contrast, conditional astroglia- and projection neuron-specific Pex5^−/−^ mice were largely asymptomatic (Kassmann et al. 2007; Bottelbergs et al. 2010). Remarkably, peroxisomes are heterogeneously distributed inside neurons and are largely absent from the axonal compartment of long projection neurons (Kassmann et al. 2007, 2011), which might explain why the corresponding knockouts did not induce axonal degeneration. By contrast, peroxisomes are highly abundant in the myelin-forming oligodendrocytes surrounding the axons. To analyse the functional cooperation between peroxisomes in myelinating cells and axons, the authors studied a conditional oligodendrocyte-specific Pex5 knockout strain (Cnp–Pex5^−/−^). In the peripheral Schwann cell-associated nerves, which are not compromised by immune-mediated injury and dysmyelination like neurons in the CNS, vesicular accumulations were observed in swellings close to the nodes of Ranvier (Kassmann et al. 2011). Such axonal swellings are a typical phenomenon preceding axonal degeneration (Griffiths et al. 1998). In line with electrophysiological dysfunctions, these axons showed an abnormal internodal localization of normally juxtaparanodally positioned membrane proteins (Kv1 channels, CASPR2, TAG-1) (Kleinecke et al. 2017). In healthy nerves, GD1 gangliosides form paranodal lipid raft-like structures required for correct membrane protein positioning. In the Cnp–Pex5^−/−^ mice, however, the myelinated nerves exhibited dispersed internodal GD1 gangliosides with increased acyl chain length. These internodal GD1 clusters partially colocalized with lysosomes suggesting that the accumulating gangliosides could not be degraded. As peroxisomes were found in close association with these lysosomal accumulations, the authors concluded that the defect in peroxisomal β-oxidation precludes the degradation of VLCFA incorporated into the gangliosides. Accordingly, gangliosides accumulate in lysosomes and cell membranes, compromising axonal transport processes, positioning of membrane proteins and ultimately nerve electrophysiology (Kleinecke et al. 2017).

While knockout of Pex5 disrupts all major peroxisomal pathways, it remains to be clarified how individual metabolic peroxisomal functions contribute to the brain pathology of patients with peroxisomal disorders. Peroxisomal β-oxidation is responsible for the degradation of straight VCLFA, branched-chain fatty acids and cholesteryl ester side chains. Therefore, it is important to identify, which metabolites may target the brain in peroxisomal disorders. In addition to the most prevalent disorder, X-linked adrenoleukodystrophy (X-ALD), which is evoked by mutations in the peroxisomal fatty acid transporter ABCD1 (Fig. 1), important SEDs with a severe brain pathology are caused by mutations in the genes of acyl-CoA oxidase 1 (ACOX1) and the multifunctional protein 2 (MFP2, encoded by HSD17B4) (Berger et al. 2016). These enzymes catalyse the first and second steps in peroxisomal β-oxidation, respectively. Moreover, isoforms with different substrate specificities exist for both proteins. ACOX1 preferentially degrades straight chain fatty acids, while ACOX2 and ACOX3 handle branched-chain fatty acids and cleave the side chains of cholesteryl esters in the pathway of bile acid synthesis (Van Veldhoven 2010). Recently, patients with a mutated, non-functional ACOX2 have been identified who show markedly elevated levels of C27 bile acid intermediates (Vilarinho et al. 2016; Monte et al. 2017). The patients suffer primarily from a liver pathology, whereas neurological functions are only mildly compromised (Vilarinho et al. 2016). Branched-chain fatty acid levels are not altered in ACOX2 patients, implying a functional complementation by ACOX3 (Ferdinandusse et al. 2018). By contrast, ACOX1 patients exhibit a mild Zellweger-like pathology with visual and hearing impairment, and degenerations in cerebral and cerebellar white matter tracts resulting in psychomotor retardation and progressive loss of motor achievements (Ferdinandusse et al. 2007). Thus, accumulation of straight VCLFAs might be especially toxic for the human brain. However, corresponding ACOX1^−/−^ mice do not develop a CNS phenotype but rather a severe liver pathology (Fan et al. 1998). Hence, other mouse models were required to investigate the role of peroxisomal β-oxidation in the brain pathology of peroxisome disorders.

MFP2 (also termed D-bifunctional protein) catalyses the second and third steps in peroxisomal β-oxidation and processes most of the metabolites emerging from step one. MFP2-deficient patients suffer from a severe brain pathology including neuronal migration defects and a progressive demyelination. With MFP1 (L-PBE), an alternative enzyme exists, which might compensate for the loss in MFP2. However, MFP1^−/−^ mice show no reduction in peroxisomal β-oxidation or a pathologic phenotype. In contrast, MFP2^−/−^ mice accumulate VLCFA, branched-chain fatty acids and bile acid intermediates in plasma and tissues and are, hence, a good model for a generally disrupted peroxisomal β-oxidation pathway (Baes et al. 2000). These mice show none of the developmental alterations observed in human MFP2 patients, but like humans develop a severe, progressive neuropathology exhibiting the first signs of dyskinesia before the age of 1 month, show a profound inflammatory pathology and usually die at an age of around 6 months (Huyghe et al. 2006). Liver peroxisomes contribute significantly to the maintenance of brain lipid homeostasis, and accordingly a liver-specific MFP2 knockout mouse developed a most severe brain phenotype (Krysko et al. 2007). It remained to be clarified, if the individual β-oxidation in the main neuronal cell types also contributes to the brain pathology. To this end, the Baes group established a nestin–MFP2^−/−^ strain ablating peroxisomal β-oxidation in all neural cell types (nestin–MFP2^−/−^), an oligodendrocyte-specific MFP2 deletion (Cnp–MFP2^−/−^) and a Purkinje cell-specific deletion (L7–MFP2^−/−^) strain (Verheijden et al. 2013; De Munter et al. 2018). The nestin–MFP2^−/−^ mouse showed the most severe pathology establishing a locomotor phenotype comparable to the constitutive MFP2 knockout. Early on the mice develop a progressive ataxia, kyphosis and abnormal limb positioning; however, they also exhibit a prolonged life and generally less severely altered neurologic parameters than the full knockout (Verheijden et al. 2013; Beckers et al. 2018). Morphologically, the neural phenotype was accompanied by cerebellar atrophy with early-onset axonal swellings and a dramatic reduction in Purkinje cells at an age of 1 year. Both, the total and the neural MFP2 knockouts developed an inflammatory brain phenotype, which, however, differed significantly in its severity (Verheijden et al. 2013; Beckers et al. 2018). The authors concluded that the initial primary neuronal deficits are exaggerated by the strong microglia activation only found in the constitutive MFP2^−/−^ mice (Beckers et al. 2018). Unexpectedly, the deletion of MFP2 from oligodendrocytes (Cnp–Pex5^−/−^) resulted in a rather mild phenotype without signs of ataxia and inflammatory responses before 12 months of age. Nevertheless, peripheral neurons exhibited the same mislocalization of juxtaparanodal membrane proteins observed for the Cnp–Pex5^−/−^ mice indicating that the accumulation of peroxisomal β-oxidation metabolites induced alterations at the molecular level (Kleinecke et al. 2017). In the light of the severe neuropathological phenotype of the oligodendrocyte-specific Pex5^−/−^ mice, these findings are intriguing and imply that the lack of β-oxidation in cerebellar oligodendrocytes can be compensated by the peroxisomes in the remaining neural cell types. In contrast, such compensation is not possible for the Purkinje cell-specific deletion in the correspondent L7–MFP2^−/−^ mice. This strain developed symptoms of ataxia already at the age of 6 months and showed a significant decline in Purkinje cell numbers at later stages (De Munter et al. 2018). According to these findings, the role of peroxisomal metabolism in the CNS appears to be more complex than previously anticipated. Obviously, the significance of peroxisomes cannot be merely associated with the individual neural cell types. Rather, peroxisomes in neurons, astrocytes and oligodendrocytes appear to perform locally distinct functions that are of differing importance in individual CNS areas.

Peroxisomal β-oxidation is also compromised in X-ALD, since the mutation of the correspondent ABC transporter ABCD1 disrupts the import of VLCFAs into peroxisomes (Engelen et al. 2014) (Fig. 1). However, it remains unclear how the accumulating VLCFAs could mechanistically induce harmful alterations in the brain tissue. Elevated lipid peroxidation products have been found in X-ALD patient plasma samples suggesting that oxidative stress might be involved in the pathogenesis (Nury et al. 2017). Findings from ABCD1^−/−^ human fibroblasts and cultured neural mouse cells revealed an enhanced generation of ROS upon VLCFA exposure suggesting that lipid-induced oxidative damage might directly contribute to the neuropathological alterations (Fourcade et al. 2008; Hein et al. 2008; Kruska et al. 2015). Increased incorporation of VLCFAs into the phospholipids of the inner mitochondrial membrane might destabilize OXPHOS complexes inducing electron leakage and ROS production, finally compromising cell physiology (López-Erauskin et al. 2013; Fourcade et al. 2014). However, disease severity did not correlate with VLCFA elevation in the different conditional MFP2^−/−^ strains (Verheijden et al. 2013, 2014). Thus, accumulation of VLCFAs might not be mainly responsible for the ROS-induced and inflammatory pathology observed in many peroxisomal disorders. Similar observations have also been reported for the different Pex5^−/−^ mouse strains (Bottelbergs et al. 2010). While these data might question a simple dose–response correlation between accumulating VLCFAs, mitochondrial ROS production and the cytopathological alterations in the brain of peroxisome disorder patients, the relation between peroxisomal dysfunction and changes in the mitochondrial redox balance remain evident. Inhibition of peroxisomal catalase in mouse embryonic fibroblasts induced changes in the mitochondrial redox equilibrium (Rahim et al. 2016). Therefore, dysregulation of peroxisomal lipid metabolism and ROS production might directly target mitochondria in the brain in peroxisome disorders (Rahim et al. 2016). Hence, while we increasingly understand the tissue pathology in peroxisomal β-oxidation disorders, one of the future challenges will be to decipher active metabolites, signalling systems and cytological alterations, which induce the severe brain pathology.

Myelin sheaths contain comparatively high concentrations of plasmalogens/ether lipids synthesized in peroxisomes (Wanders and Poll-The 2017). Thus, it is not surprising that a lack in ether lipid synthesis induces CNS pathology. The peroxisomal disorder rhizomelic chondrodysplasia punctata (RCDP) is caused by a disrupted ether lipid synthesis pathway. RCDP types 2–4 are peroxisomal SEDs which are caused by mutations in the genes for dihydroxyacetone phosphate acyltransferase (GNPAT), alkyl-dihydroxyacetone phosphate synthase (ADHAPS) and fatty acyl-CoA reductase 1 (FAR1) (Dorninger et al. 2017) (Fig. 1). With respect to the CNS pathology, RCDPs are characterized by myelination deficits, which result in enlarged ventricles and subarachnoidal spaces, as well as cerebellar atrophy (Dorninger et al. 2017). To analyse the molecular pathogenesis, two mouse models with a deletion in GNPAT and ADHAPS have been generated (Rodemer et al. 2003; Liegel et al. 2014). GNPAT^−/−^ mice develop neuropathological symptoms typical for RDCP such as a general reduction in hemisphere size, foliation defects of the cerebellum or a reduced myelination in the CNS white matter (Teigler et al. 2009). At the subcellular level, the mice show changes in Purkinje cells, like alterations in the synaptic innervation pattern from parallel and climbing fibres in dendrites as well as axonal swellings, which are paralleled by a disorganization in paranodal membrane proteins (Teigler et al. 2009). Berger and coworkers investigated the influence of the ether lipid deficiency on presynaptic functions (Brodde et al. 2012). In parallel to a reduced Ca^2+^-dependent neurotransmitter release the authors report a reduction in the respiratory capacity of synaptic mitochondria. The lack of plasmalogens in mitochondrial membranes might disturb OXPHOS complexes and thus ATP generation by the mitochondrial electron transport chain. Since release and regeneration of synaptic vesicles are ATP-dependent processes, mitochondrial dysfunction would eventually compromise synaptic transmitter release. In addition to the CNS pathology, GNPAT^−/−^ mice exhibit impaired axonal sorting and myelination in PNS sciatic nerves, which were ascribed to a dysregulation in p-AKT/GSK3β signalling in Schwann cells (da Silva et al. 2014; Hossain et al. 2017). Remarkably, GSK3β activity was reported to modulate Schwann cell differentiation and initiation of myelination (Ogata et al. 2004), which might explain the myelination defects observed in the GNPAT^−/−^ mice.

As documented by the brain pathology in both β-oxidation as well as plasmalogen deficiencies, correct lipid homeostasis appears to be a crucial factor for brain physiology. During the last years, our understanding of the cytopathological alterations observed in the brain in peroxisomal disorders revealed that different cell types in different brain areas may contribute to disease pathogenesis. Moreover, we gained important knowledge on the distinct pathology of individual SEDs. However, our understanding of the underlying molecular mechanisms leading from metabolic changes to the severe cytological alterations in the brain is still scarce and requires future research.

In addition to the developments in the field of peroxisomal disorders, peroxisome alterations have been recently associated with the pathogenesis of more widespread neurological disorders (Berger et al. 2016). Increased VLCFAs as well as decreased plasmalogen concentrations were observed in cortical brain regions of advanced Alzheimer patients (Kou et al. 2011). The decrease in plasmalogens in Alzheimer patients and respective Alzheimer mouse models was further corroborated by more recent studies (Dorninger et al. 2017). Moreover, inhibition of peroxisomal β-oxidation was reported to increase the amount of Aβ generation in rat brain (Shi et al. 2012). These findings could indicate that a dysregulation in peroxisomal lipid metabolism might contribute to Alzheimer pathogenesis.

Organelle transport defects are a common observation in neurodegenerative diseases as cellular transport systems have to ensure correct organelle distribution and removal inside the highly polarized neurons (De Vos and Hafezparast 2017). Increased peroxisome volume densities were detected in the somata of neurons from patients with a pronounced Alzheimer pathology. In contrast, peroxisomes were absent in their neuronal processes when these were positive for the Alzheimer pre-tangle marker-phosphorylated tau (Kou et al. 2011). Thus, efficient peroxisome transport between neurites and somata might be compromised in Alzheimer-affected neurons at an early stage in neuronal degeneration.

Peroxisome alterations were also reported in transgenic mouse models for Alzheimer’s disease (Cimini et al. 2009; Fanelli et al. 2013). However, it remains to be determined if the changes in peroxisome metabolites are a secondary phenomenon or play a causative role in the disease pathogenesis. In either case, the changes in the neuronal lipid composition might aggravate disease progression and thereby contribute to Alzheimer pathology.

While there are still limited amount of data on the peroxisome contribution in Alzheimer disease, first studies targeting peroxisomes for therapeutic issues have already been performed. Treatment with peroxisome proliferators was reported to mitigate spatial memory impairment, synaptic failure, and neurodegeneration in transgenic Alzheimer model mice (Inestrosa et al. 2013). However, as the transcriptional response to peroxisome proliferators includes numerous non-peroxisomal genes, further studies are required to substantiate that a specific stimulation of peroxisomal functions was responsible for the observed effects. Furthermore, oral substitution therapy has been considered as a therapy to restore normal plasmalogen levels in patients. Oral administration of purified scallop-derived plasmalogen was reported to improve cognitive functions in mild Alzheimer patients (Fujino et al. 2017). However, systematic animal studies imply that plasmalogens and their respective precursors do not efficiently cross the blood–brain barrier and are not incorporated into the CNS (Dorninger et al. 2018). Therefore, until further proof is provided, the value of an oral plasmalogen replacement therapy should be regarded with great caution.

In Parkinson’s disease, the presynaptic protein α-synuclein aggregates to form intracellular, insoluble, filamentous Lewy bodies, a cytopathological hallmark of the disease (Spillantini et al. 1997). Before Lewy body formation, several posttranslational modifications like phosphorylation accumulate in α-synuclein and are regarded as early events in Parkinson pathology (Barrett and Timothy Greenamyre 2015). Interestingly, Pex2^−/−^, Pex5^−/−^, and Pex13^−/−^ mouse models exhibit increased α-synuclein phosphorylation, oligomerization and inclusion body formation (Yakunin et al. 2010). Long-chained, unsaturated fatty acids have been described as inducing factors for α-synuclein aggregation (Sharon et al. 2003; Assayag et al. 2007). In this regard, the accumulation of polyunsaturated long-chain fatty acids in Pex-deficient mutants might suggest a link between peroxisome dysfunction and Parkinson’s disease (Yakunin et al. 2010).

Compromised peroxisome physiology was recently described in the brain grey matter from multiple sclerosis (MS) patients using ABCD3 immunocytochemistry and gene expression analysis (Gray et al. 2014). In parallel, the authors observed elevated VLCFA concentrations in the affected brain regions suggesting a decline in peroxisomal metabolism. In line with these observations, decreased enzyme activities and gene expression for peroxisomal proteins were also reported in the CNS of a mouse model for MS with inflammatory autoimmune encephalomyelitis (Singh et al. 2004). Hence, peroxisome abundance might decrease during MS progression and activation of peroxisome biogenesis might be favourable in MS and other demyelinating disorders. Statins, in addition to their cholesterol-lowering effect, possess anti-inflammatory and immune-modulatory activities (Stanislaus et al. 2002; Vollmer et al. 2004). These responses are mediated by induction of PPARα/γ signalling pathways thus inducing peroxisome proliferation (Paintlia et al. 2013). However, statin therapy had only limited efficacy on the CNS in MS (Pihl-Jensen et al. 2015). The AMPK activator 5-aminoimidazole-4-carboxamide ribonucleotide (AICAR) was also reported to reduce pro-inflammatory and immune responses in experimental autoimmune encephalomyelitis (Nath et al. 2009). A combined administration of the statin lovastatin and AICAR to autoimmune encephalomyelitis mice alleviated inflammation-induced dysfunction of mitochondria and peroxisomes as well as demyelination. Thus, restoring peroxisome function might be beneficial for MS disease prognosis (Singh et al. 2018).

Ischemic stroke is one of the major causes of death in modern societies. Understanding neuroprotective mechanisms associated with cerebral ischemia is a prerequisite for the development of future therapeutic inventions. In mice, peroxisome proliferation was observed after focal cerebral ischemia induced by middle cerebral artery occlusion suggesting a protective response (Young et al. 2015). For mechanistic studies, the authors induced ischemic injury in cortical neuron cultures by oxygen–glucose deprivation, and similar to the in vivo situation, neurons responded with peroxisome proliferation. When peroxisome division was impeded by Drp1-knockdown or when catalase activity was inhibited with 3-amino-1,2,4-triazole, increased neuronal cell death was observed in response to the ischemic insult. In contrast, administration of PPARα agonists had a protective effect on neuron survival rates (Young et al. 2015). The authors concluded that peroxisomes might exhibit protective functions against oxidative or metabolic stress induced after ischemia–reperfusion injury which might be targeted as therapy for neuroprotection after stroke.

In summary, the current literature implies that peroxisome abundance and metabolism play a role in a variety of pathologic states of the brain; however, it remains unclear if these peroxisome alterations primarily contribute to the pathogenesis of the disease described above or if they are secondary changes associated with a general decline in cellular functions during disease progression. Therefore, further studies are required, which systematically analyse peroxisome alterations at different disease stages, especially to unravel if therapeutic strategies targeting peroxisomes might be relevant to combat disease progression.

## Peroxisomes in the auditory system: mysterious hearing loss

Progressive hearing loss is one of the typical pathologies associated with inherited peroxisomal disorders (Braverman et al. 2016), thus pointing to an important functional role of peroxisomes in the auditory system. Pejvakin (Persian word for echo)-deficient humans and mice show a striking hyper-vulnerability towards sound exposure. Pejvakin^−/−^ mice show features of marked oxidative stress and impaired antioxidant defences in hair cells and cochlear ganglion neurons (Delmaghani et al. 2015). Subcellular localization studies with an antibody raised against the C-terminal region of pejvakin showed that endogenous pejvakin localizes to peroxisomes of inner ear hair cells, and pejvakin^−/−^ mice revealed peroxisome abnormalities in shape and localization after onset of hearing (Delmaghani et al. 2015). Expression of wild-type and mutant pejvakin resulted in increased or decreased peroxisome proliferation suggesting that pejvakin is involved in peroxisome biogenesis. In line with these findings, peroxisome proliferation was observed in inner and outer hair cells as well as dendrites of primary auditory neurons in response to sound exposure, whereas pejvakin^−/−^ mice exhibited decreased peroxisome numbers (Delmaghani et al. 2015). A more recent publication, however, doubted the peroxisomal localization of pejvakin (Kazmierczak et al. 2017). No colocalization of expressed pejvakin and the peroxisomal membrane marker PMP70 was observed in HeLa cells using three different polyclonal antibodies designed against alternative pejvakin peptide sequences. Likewise, expression of pejvakin in inner hair cells did not result in a peroxisomal staining but selectively localized to stereociliary rootlets (Kazmierczak et al. 2017). Thus, while there is some evidence that peroxisomes react to sound exposure, potentially to counteract intracellular ROS generation, the role of pejvakin in peroxisome physiology remains uncertain.

## Peroxisomes and cancer: a mysterious connection

Cancer cells face a completely different microenvironment than normal cells and have therefore to adapt their cellular metabolism to the hypoxic and hypo-nutrient conditions in a tumor (Yoshida 2015). This process of metabolic reprogramming is considered one of the major hallmarks of cancer and in addition to changes in glucose and amino acid metabolism, alterations in lipid metabolism have been reported (Ghaffari et al. 2015). Moreover, dysregulations in cellular redox homeostasis can not only be pro-tumorigenic but also lead to resistance in tumor chemotherapy (Glasauer and Chandel 2014). Mitochondria have been considered one of the key organelles for these alterations in metabolism and redox homeostasis (Valcarcel-Jimenez et al. 2017; Ježek et al. 2018) and as mitochondrial and peroxisomal functions are closely linked (Schrader et al. 2015a, b), peroxisome physiology might also be relevant in the process of the transition of somatic into tumor cells.

Peroxisomes, housing a variety of oxidases, are potent H_2_O_2_ producers and an imbalance in H_2_O_2_ generation and degradation during the PPARα-mediated induction of peroxisomal fatty acid β-oxidation has been linked to the occurrence of liver tumors during chronic exposure to peroxisome proliferating drugs (Yu et al. 2003). Nevertheless, studies investigating the role of peroxisomes in spontaneously occurring tumor tissue remain limited. In a significant number of neoplastic tissues investigated, peroxisomal function appears to decline when compared to unaffected tissue. A reduced number of peroxisomes was observed in colon carcinomas using electron microscopy to detect catalase by alkaline DAB staining (Cablé et al. 1992). These data were corroborated by several publications reporting reduced peroxisomal protein abundance (catalase, ABCD3, ACOX1, PXMP2) or enzymatic activities (catalase, D-amino acid oxidase, polyamine oxidase, peroxisomal β-oxidation) in colon tumor tissue, implying an overall reduction of peroxisome abundance and function in the neoplastic tissue (Baur and Wendel 1980; Cablé et al. 1992; Keller et al. 1993; Lauer et al. 1999). Likewise, a reduction in peroxisomal enzyme activities or protein amounts was reported in breast and hepatocellular carcinomas, respectively (Keller et al. 1993; Litwin et al. 1999). Interestingly, Lauer et al. (1999) observed increased mRNA levels of the correspondent enzymes suggesting an imbalance in the turnover of peroxisomes which could be explained by incompetence in peroxisome biogenesis or increased rates of peroxisome degradation (pexophagy). However, conflicting data were published in a more recent study, which reports that peroxisomes are indispensable for the survival of liver cancer cells (Cai et al. 2018). As previous publications observed an up-regulation of Pex2 at the mRNA level in hepatic carcinomas (Chen et al. 2002; Wurmbach et al. 2007), Cai and colleagues silenced Pex2 by RNAi in hepatocellular carcinoma xenografts and reported significantly reduced tumor growth in response to the treatment. Furthermore, the authors report that the loss in peroxisome function leads to increased ROS levels by mislocalization of catalase to the cytosol. Subsequent ER stress in the tumor cells would result in suppression of mTORC1 signalling and elevation of autophagy ultimately leading to cell death (Cai et al. 2018). Contradicting these interpretations, recent publications showed that a reduced catalase import rate is a cellular mechanism to protect cells from redox stress (Fujiki et al. 2017; Walton et al. 2017). Recently published data on the potential role of ACOX1, the rate limiting and H_2_O_2_-generating oxidase in peroxisomal β-oxidation, in hepatocellular tumors underlines the complexity in understanding peroxisomal function in hepatocarcinogenesis (Chen et al. 2018). In this study, ACOX1 enzyme activity was found to be decreased by SIRT5-dependent lysine de-succinylation. After SIRT5 knockdown, the authors consequently observed an increase in intracellular H_2_O_2_ levels. Interestingly, the authors report that while ACOX1 protein levels were comparable or slightly reduced if compared to surrounding liver tissue, ACOX1 activity was elevated, and SIRT5 expression decreased in most of the tumor samples. According to these findings increased ACOX1 succinylation might lead to excess H_2_O_2_ generation in the tumor cells thereby promoting the transformation of healthy into tumor cells.

In the healthy kidney, peroxisomes are most numerous in the epithelial cells of the proximal tubule (Islinger et al. 2010a, b). In renal clear cell tumors, which recapitulate the tissue from proximal tubules (Prasad et al. 2007), however, peroxisomes were reported to be absent according to alkaline DAB and immunofluorescence detection of catalase (Frederiks et al. 2010). In agreement with this observation, decreased catalase activities were found in renal tumors (Pljesa-Ercegovac et al. 2008). The hypoxia-inducible transcription factor Hif2a has been recently shown to promote peroxisome degradation via autophagy (Walter et al. 2014). Remarkably, the authors showed that Hif2a levels negatively correlated with the abundance of peroxisomes in renal clear cell carcinoma, suggesting that its induction in a hypoxic tumor environment depletes peroxisomes by enhanced pexophagy rates. Nevertheless, as all current findings on peroxisome abundance in renal tumors rely on catalase detection, future studies applying additional peroxisome markers have to verify a decrease in peroxisome abundance. When summarized, these publications imply that a disruption of the peroxisomal compartment could be a major general hallmark in cancer biology. A reduction in peroxisomes might lead to local alterations in membrane lipid composition thereby altering the integration of neoplastic cells into the surrounding tissue. In addition, the loss of peroxisomes might destabilize the intracellular ROS equilibrium and lead to locally elevated toxic, and partially oxidized VLCFA metabolites. In this respect, it is worthwhile to note that the latter were hypothesized to induce an ER-stress response in ACOX1 knockout mice, which may eventually trigger the formation of liver tumors, which are regularly observed in this mouse line (Huang et al. 2011).

While the publications described above point to a general loss of peroxisome function during tumor progression, findings from prostate cancer tissue implement a more complex role of peroxisomes in the maintenance of tumor growth. The peroxisomal α-methylacyl-CoA racemase (AMACR) expression was found to be highly elevated in tissue from prostate carcinoma if compared to benign prostate tissue (Jiang et al. 2001). Since this initial publication, numerous studies have confirmed elevated levels of AMACR as a reliable prostate cancer tumor marker (Lloyd et al. 2008). AMACR is an accessory enzyme in the α-oxidation pathway for phytanic acid and required for the conversion of 2R-methylacyl-CoA into 2S-methylacyl-CoA (Wanders and Waterham 2006). 2S-Pristanoyl-CoA is further degraded via the peroxisomal β-oxidation pathway for 2-methyl branched-chain fatty acids. Subsequent studies have revealed that expression of other enzymes involved in the peroxisomal branched-chain fatty acid degradation pathway (e.g. ACOX3, D-PBE, and the 3-ketoacyl-CoA thiolase ACAA1) is increased in prostate tumors (Zha et al. 2005; Valença et al. 2015). Moreover, the monocarboxylate transporter 2 (MCT2) (Fig. 1) was recently shown to reallocate in peroxisomal membranes of malignant prostate cancer cells (Valença et al. 2015). Monocarboxylate transporters could be responsible for the shuttling of lactate–pyruvate to re-oxidize NADH to regenerate NAD^+^ as a cofactor for peroxisomal β-oxidation (McClelland et al. 2003). These findings might point to a specific elevation of peroxisomal branched-chain fatty acid metabolism in prostate tumors. Interestingly, elevated AMACR expression was also reported from colon, gastric, breast, renal and hepatocellular carcinoma (Jiang et al. 2003; Witkiewicz et al. 2005; Chen et al. 2005; Went et al. 2006; Jindal et al. 2016) suggesting that peroxisomal branched-chain metabolism might be associated with a broader variety of tumors. In this regard, it is tempting to speculate why the degradation of branched-chain fatty acids might be elevated in prostate and other tumors. One possibility is a correlation between elevated serum phytanic acid levels, which could lead to the induction of AMACR, and the occurrence of prostate cancer, which has been reported (Xu et al. 2005) but is currently under debate (Kataria et al. 2015). Moreover, a reduction in AMACR expression by RNAi has been reported to reduce the growth rates of the prostate cancer cell line LAPC-4 (Zha et al. 2003). Human AMACR deficiency is represented by variable phenotypes including childhood cholestasis, late-onset peripheral neuropathy, pigmentary retinopathy or seizures but is not associated with increased tumor development (Ferdinandusse et al. 2000). These findings suggest that metabolites downstream of AMACR might be the active compounds triggering cancer development, e.g. by activating receptors of relevant signalling pathways such as PPARs or RXRs. On the other hand, more general alterations in lipid metabolism have been proposed to interfere with the tumor development (Wu et al. 2014). To substantiate the latter, data on the levels of further enzymes and lipid metabolites associated with peroxisomal, mitochondrial and ER metabolism would be required to increase our insights into the pathology of prostate cancer. In addition to the enzymes of the peroxisomal branched-chain fatty metabolism, the peroxisomal membrane protein PMP24/PXMP4 has been associated with the development of prostate cancer. In contrast to the former, however, PMP24 has been reported to be silenced by methylation of a single intronic CpG during the transition of the prostate cancer cell line LNCaP from androgen dependence to androgen independence (Wu and Ho 2004; Zhang et al. 2010). PMP24 is a member of the TIM17 family of membrane proteins (Fig. 1) but its function is currently unknown. Nevertheless, the findings indicate that peroxisomes might be involved at different stages during the transition of healthy prostate tissue into malignant cancer cells.

Peroxisomes in glioblastomas have been investigated with respect to tumor grade progression (Benedetti et al. 2010). In correlation with the progressing tumor grade an increasing staining for peroxisomes was observed using immunocytochemistry. These findings were validated by detection of the proteins Pex14, PMP70, ACOX1, and 3-ketothiolase using immunoblotting, which indicate peroxisome proliferation leading to increased organelle numbers. In parallel, the tumors showed multiple lipid droplets and an elevated expression of PPARα. In a follow-up study, the authors investigated the influence of hypoxic conditions on peroxisomes in primary cultures of *Glioblastoma multiforme* (GBM) tumors (Laurenti et al. 2011). According to PMP70 immunofluorescence staining, an increase in peroxisomes and lipid droplets was reported. In parallel, an induction of the hypoxia-inducible factor Hif1α and PPARα was observed in response to hypoxia. The authors concluded that Hif1α activation under the hypoxic conditions in a tumor might induce PPARα expression, which subsequently triggers peroxisome proliferation. Nevertheless, it remains to be clarified why PPARα-induction, which generally induces catabolic lipid metabolism, correlates with the increased abundance of lipid droplets in the GBM cells. After observing an increase in HMG-CoA reductase as well as cholesterol and triglyceride levels in the cultures, the authors hypothesized that peroxisomal β-oxidation under hypoxic conditions might be used to produce acetyl-CoA as a substrate for de novo lipid synthesis. Another recently identified peroxisomal protein, HSDL2 (hydroxysteroid dehydrogenase-like 2) also appears to be up-regulated in glioblastomas (Ruokun et al. 2016) and ovarian cancer (Sun et al. 2018). Knockdown of HSDL2 resulted in decreased growth rates in glioblastoma cell lines, and inhibited cell proliferation, colony formation, motility, and tumorigenesis in ovarian cancer cells underlining an important role for peroxisomes in these tumor types. At the current stage, further data are required to decipher if peroxisomes play a role in the metabolic transformation of glial cells into malignant glioblastoma.

Peroxisomes are dynamic organelles which are able to adapt their number and enzyme content to the specific requirements of their cellular environment (Schrader et al. 2015). Therefore, it has to be considered that drugs applied in therapeutic cancer intervention might remodel peroxisomes thereby influencing tumor physiology. Dahabieh and colleagues investigated the reaction of peroxisomes to Vorinostat, a HDAC inhibitor used for lymphoma treatment, which promotes ROS-mediated apoptosis to evaluate their potential role in resistance to tumor intervention (Dahabieh et al. 2017). The study revealed that peroxisomes in cultured lymphoma cells were indeed up-regulated in response to Vorinostat administration. Consequently, knockdown of Pex3 or more specifically catalase significantly increased ROS-mediated apoptosis in the lymphoma cells in response to Vorinostat treatment. Thus, peroxisomes, which are ROS-degrading organelles, have to be considered to play a role in resistance to therapeutic tumor intervention when drugs inducing ROS-mediated apoptosis are applied.

Wu and coworkers observed an increased expression of the peroxisomal Lon protease LonP2 in cervical cancer tissue (Wu et al. 2018). Functionally, LonP2 fulfils the function of a combined chaperone/protease, refolding or degrading compromised peroxisomal proteins (Bartoszewska et al. 2012). A down-regulation of LonP2 in the tumor cell lines HeLa and SiHA reduced oxidative stress and inhibited cervical cancer cell proliferation and migration (Wu et al. 2018). As the study, however, lacks any further analysis on the peroxisomal status in the cervical cancer cells or LonP2 depleted cell lines, it is currently impossible to mechanistically explain how the changes in LonP2 expression might modify peroxisome physiology with respect to cancerogenesis.

Most of the studies described above focus on the metabolic role of peroxisomes in tumor development. Interestingly, a recent publication might add an unexpected function of peroxisomes in the control of correct cell division (Asare et al. 2017). The study reveals that correct peroxisome positioning during mitosis is required for the correct asymmetric cell division in skin epithelial cells. RNAi-mediated knockdown of Pex11β and Pex14 resulted in mitotic delay in the targeted cells and led to an imbalance in growth and differentiation into basal and supra-basal skin cells accompanied by a reduction in terminal differentiation markers in the tissue. Remarkably, the mitotic dysregulation was not associated with a disruption in peroxisomal metabolic functions but was found to be caused by a mislocalization of peroxisomes during spindle formation. Under normal conditions, peroxisomes are positioned at the spindle poles whereas a deviating localization resulted in uncontrolled spindle rotations and triggered an arrest at a mitotic check point for organelle segregation (Asare et al. 2017). Summarizing their results the authors concluded that proper peroxisome inheritance has a role in controlling the balance between cell growth and differentiation. Perturbations in this system lead to the generation of basal daughter cells with differentiation markers but still proliferating features typically associated with cancer (Asare et al. 2017).

In summary, our current view on the role of peroxisomes in cancer cells remains fragmentary and while earlier publications mainly reported a decrease in peroxisome activity in many tumors, more recent studies also suggest that specific peroxisome functions are required for efficient tumor growth. These cursorily contradictory findings might result from the vast heterogeneity of tumors analysed. Moreover, a single tumor itself does not represent a single cell type, further complicating the interpretation of results. To substantiate our current understanding on the status of peroxisomes in tumors, extensive comparative studies would be required to associate potential peroxisome dysfunction with tumor type and grade as well as the metabolic status of the tissue.

## Concluding remarks

During the last 6 years, following the publication of our second ‘‘mystery’’ review (Islinger et al. 2012a, b), the view on peroxisome biology has further widened, adding new important discoveries in the areas of peroxisome function, biogenesis, formation, division and motility, and unveiled new proteins and machineries at the peroxisomal membrane and further insights into peroxisome–organelle interaction and cooperation. With regard to protein import, new peroxins and alternative import pathways have been identified and progress has been made in the understanding of the export and recycling of the ubiquitinated import receptors Pex5 and Pex7 via the Pex1/Pex6 complex. Concerning the process of peroxisome formation, several unexpected observations in different model organisms have given new mechanistic twists such as indirect targeting of PMPs, ER- and mitochondria-driven pre-peroxisomal vesicle formation and de novo formation of peroxisomes. This resulted in a more complex model of peroxisome formation, and a challenge in the field is to build an overall understanding of the general process (reviewed in Costello and Schrader 2018). The discovery of new peroxisome–organelle contact sites and molecules involved in tethering has broadened our thinking on peroxisome cooperation and crosstalk with other compartments, in particular, with respect to associated diseases, where the role and importance of contact sites are only now starting to be revealed. It will be a challenge for the future to develop techniques to identify the proteins that mediate contacts and metabolic channelling, especially those transferring lipids from the ER to peroxisomes for membrane expansion and division. The field of membrane contacts and organelle cooperation is just in its infancy, and new contact sites, components, functions, and regulators await discovery. It is also now evident that peroxisomes are key metabolic organelles with protective functions and a wider significance in human health with potential impact on a large number of globally important human diseases such as neurodegenerative disorders, obesity, cancer, and diabetes (Elsner et al. 2011; Zhou et al. 2018). Further systematic studies are required to validate if peroxisome alterations/dysfunctions primarily contribute to the disease aetiology or if these are secondary changes reflecting a general decline in cellular fitness with disease progression. In addition, the functional correlation between disease pathogenesis and alterations in peroxisome physiology has to be deciphered. Other rapidly developing research areas include the role of peroxisomes in cellular redox balance and redox signalling (Fransen and Lismont 2018) and in antiviral signalling and defence (Wong et al. 2018). Peroxisomes are still among the more mysterious subcellular compartments in eukaryotic cells, but there is no doubt that they are “on the rise” and poised to reveal more surprises in the near future.

## Abbreviations

ACBD: Acyl-CoA-binding domain
ACOX: Acyl-CoA oxidase
CNS: Central nervous system
EE: Early endosome
ER: Endoplasmic reticulum
MFP: Multifunctional protein
MS: Multiple sclerosis
NO: Nitric oxide
PBD: Peroxisome biogenesis disorder
Pex: Peroxin
PMP: Peroxisomal membrane protein
PPAR: Peroxisome peroxisome-proliferator receptor
PTS: Peroxisomal targeting signal
ROS: Reactive oxygen species
SED: Single enzyme deficiency
VLCFA: Very long chain fatty acids
X-ALD: X-linked adrenoleukodystrophy

## References

[CR1] Agrawal G, Subramani S (2016). De novo peroxisome biogenesis: evolving concepts and conundrums. Biochim Biophys Acta Mol Cell Res.

[CR2] Ahlemeyer B, Neubert I, Kovacs WJ, Baumgart-Vogt E (2007). Differential expression of peroxisomal matrix and membrane proteins during postnatal development of mouse brain. J Comp Neurol.

[CR3] Angermüller S (1989). Peroxisomal oxidases: cytochemical localization and biological relevance. Prog Histochem Cytochem.

[CR4] Angermüller S, Fahimi HD (1988). Heterogenous staining of D-amino acid oxidase in peroxisomes of rat liver and kidney. A light and electron microscopic study. Histochemistry.

[CR5] Asare A, Levorse J, Fuchs E (2017). Coupling organelle inheritance with mitosis to balance growth and differentiation. Science.

[CR6] Assayag K, Yakunin E, Loeb V (2007). Polyunsaturated fatty acids induce alpha-synuclein-related pathogenic changes in neuronal cells. Am J Pathol.

[CR7] Aung K., Hu J. (2011). The Arabidopsis Tail-Anchored Protein PEROXISOMAL AND MITOCHONDRIAL DIVISION FACTOR1 Is Involved in the Morphogenesis and Proliferation of Peroxisomes and Mitochondria. The Plant Cell.

[CR8] Baes M, Huyghe S, Carmeliet P (2000). Inactivation of the peroxisomal multifunctional protein-2 in mice impedes the degradation of not only 2-methyl-branched fatty acids and bile acid intermediates but also of very long chain fatty acids. J Biol Chem.

[CR9] Baker DJ, Wijshake T, Tchkonia T (2011). Clearance of p16Ink4a-positive senescent cells delays ageing-associated disorders. Nature.

[CR10] Barrett PJ, Timothy Greenamyre J (2015). Post-translational modification of α-synuclein in Parkinson׳s disease. Brain Res.

[CR11] Bartoszewska M, Williams C, Kikhney A (2012). Peroxisomal proteostasis involves a Lon family protein that functions as protease and chaperone. J Biol Chem.

[CR12] Baur G, Wendel A (1980). The activity of the peroxide-metabolizing system in human colon carcinoma. J Cancer Res Clin Oncol.

[CR13] Beckers L, Stroobants S, D’Hooge R, Baes M (2018). Neuronal dysfunction and behavioral abnormalities are evoked by neural cells and aggravated by inflammatory microglia in peroxisomal β-oxidation deficiency. Front Cell Neurosci.

[CR14] Benedetti E, Galzio R, Laurenti G (2010). Lipid metabolism impairment in human gliomas: expression of peroxisomal proteins in human gliomas at different grades of malignancy. Int J Immunopathol Pharmacol.

[CR15] Benjamin DI, Cozzo A, Ji X (2013). Ether lipid generating enzyme AGPS alters the balance of structural and signaling lipids to fuel cancer pathogenicity. Proc Natl Acad Sci USA.

[CR16] Berger J, Dorninger F, Forss-petter S, Kunze M (2016). Peroxisomes in brain development and function. BBA Mol Cell Res.

[CR17] Binns D, Januszewski T, Chen Y (2006). An intimate collaboration between peroxisomes and lipid bodies. J Cell Biol.

[CR18] Blok NB, Tan D, Wang RY-R (2015). Unique double-ring structure of the peroxisomal Pex1/Pex6 ATPase complex revealed by cryo-electron microscopy. Proc Natl Acad Sci.

[CR19] Bonekamp N, Sampaio P, de Abreu FV (2012). Transient complex interactions of mammalian peroxisomes without exchange of matrix or membrane marker proteins. Traffic.

[CR20] Bottelbergs A, Verheijden S, Hulshagen L (2010). Axonal integrity in the absence of functional peroxisomes from projection neurons and astrocytes. Glia.

[CR21] Braschi E, Goyon V, Zunino R (2010). Vps35 mediates vesicle transport between the mitochondria and peroxisomes. Curr Biol.

[CR22] Braverman NE, Raymond GV, Rizzo WB (2016). Peroxisome biogenesis disorders in the Zellweger spectrum: an overview of current diagnosis, clinical manifestations, and treatment guidelines. Mol Genet Metab.

[CR23] Brodde A, Teigler A, Brugger B (2012). Impaired neurotransmission in ether lipid-deficient nerve terminals. Hum Mol Genet.

[CR24] Brown MS, Goldstein JL (1986). A receptor-mediated pathway for cholesterol homeostasis. Science.

[CR25] Brown FR, McAdams AJ, Cummins JW (1982). Cerebro-hepato-renal (Zellweger) syndrome and neonatal adrenoleukodystrophy: similarities in phenotype and accumulation of very long chain fatty acids. Johns Hopkins Med J.

[CR26] Buentzel J, Vilardi F, Lotz-Havla A (2015). Conserved targeting information in mammalian and fungal peroxisomal tail-anchored proteins. Sci Rep.

[CR27] Cablé S, Keller JM, Colin S (1992). Peroxisomes in human colon carcinomas. A cytochemical and biochemical study. Virchows Arch B Cell Pathol Incl Mol Pathol.

[CR28] Cai M, Sun X, Wang W (2018). Disruption of peroxisome function leads to metabolic stress, mTOR inhibition, and lethality in liver cancer cells. Cancer Lett.

[CR29] Campisi J, Robert L, Robert L, Fulop T (2014). Cell senescence: role in aging and age-related diseases. Interdisciplinary topics in gerontology and geriatrics.

[CR30] Carstea ED, Morris JA, Coleman KG (1997). Niemann–Pick C1 disease gene: homology to mediators of cholesterol homeostasis. Science.

[CR31] Castro IG, Richards DM, Metz J (2018). A role for Mitochondrial Rho GTPase 1 (MIRO1) in motility and membrane dynamics of peroxisomes. Traffic.

[CR32] Castro IG, Schuldiner M, Zalckvar E (2018). Mind the organelle gap—peroxisome contact sites in disease. Trends Biochem Sci.

[CR33] Cepińska MN, Veenhuis M, van der Klei IJ, Nagotu S (2011). Peroxisome fission is associated with reorganization of specific membrane proteins. Traffic.

[CR34] Chang T-Y, Chang CCY, Ohgami N, Yamauchi Y (2006). Cholesterol sensing, trafficking, and esterification. Annu Rev Cell Dev Biol.

[CR35] Chang C-R, Manlandro CM, Arnoult D (2010). A lethal de novo mutation in the middle domain of the dynamin-related GTPase Drp1 impairs higher order assembly and mitochondrial division. J Biol Chem.

[CR36] Chao Y-H, Robak LA, Xia F (2016). Missense variants in the middle domain of DNM1L in cases of infantile encephalopathy alter peroxisomes and mitochondria when assayed in Drosophila. Hum Mol Genet.

[CR37] Chen X, Cheung ST, So S (2002). Gene expression patterns in human liver cancers. Mol Biol Cell.

[CR38] Chen Z-ME, Ritter JH, Wang HL (2005). Differential expression of alpha-methylacyl coenzyme A racemase in adenocarcinomas of the small and large intestines. Am J Surg Pathol.

[CR39] Chen Y, Pieuchot L, Loh RA (2014). Hydrophobic handoff for direct delivery of peroxisome tail-anchored proteins. Nat Commun.

[CR40] Chen H, Ren S, Clish C (2015). Titration of mitochondrial fusion rescues Mff-deficient cardiomyopathy. J Cell Biol.

[CR41] Chen X-F, Tian M-X, Sun R-Q (2018). SIRT5 inhibits peroxisomal ACOX1 to prevent oxidative damage and is downregulated in liver cancer. EMBO Rep.

[CR42] Chu B-B, Liao Y-C, Qi W (2015). Cholesterol transport through lysosome-peroxisome membrane contacts. Cell.

[CR43] Cimini A, Moreno S, D’Amelio M (2009). Early biochemical and morphological modifications in the brain of a transgenic mouse model of Alzheimer’s disease: a role for peroxisomes. J Alzheimers Dis.

[CR44] Ciniawsky S, Grimm I, Saffian D (2015). Molecular snapshots of the Pex1/6 AAA + complex in action. Nat Commun.

[CR45] Cohen Y, Klug YA, Dimitrov L (2014). Peroxisomes are juxtaposed to strategic sites on mitochondria. Mol Biosyst.

[CR46] Collado M, Blasco MA, Serrano M (2007). Cellular senescence in cancer and aging. Cell.

[CR47] Corpas FJ, Barroso JB (2014). Peroxynitrite (ONOO-) is endogenously produced in arabidopsis peroxisomes and is overproduced under cadmium stress. Ann Bot.

[CR48] Corpas FJ, Barroso JB, Carreras A (2004). Cellular and subcellular localization of endogenous nitric oxide in young and senescent pea plants. Plant Physiol.

[CR49] Corpas FJ, Barroso JB, Palma JM, Rodriguez-Ruiz M (2017). Plant peroxisomes: a nitro-oxidative cocktail. Redox Biol.

[CR50] Costa A, Drago I, Behera S (2010). H2O2 in plant peroxisomes: an in vivo analysis uncovers a Ca(^2+^)-dependent scavenging system. Plant J.

[CR51] Costello JL, Schrader M (2018). Unloosing the Gordian knot of peroxisome formation. Curr Opin Cell Biol.

[CR52] Costello JL, Castro IG, Camões F (2017). Predicting the targeting of tail-anchored proteins to subcellular compartments in mammalian cells. J Cell Sci.

[CR53] Costello JL, Castro IG, Hacker C (2017). ACBD5 and VAPB mediate membrane associations between peroxisomes and the ER. J Cell Biol.

[CR54] Costello JL, Castro IG, Schrader TA (2017). Peroxisomal ACBD4 interacts with VAPB and promotes ER-peroxisome associations. Cell Cycle.

[CR55] Costello JL, Passmore JB, Islinger M, Schrader M, del Rio LA, Schrader M (2018). Multi-localized proteins: the peroxisome–mitochondria connection. Subcellular biochemistry.

[CR56] da Silva TF, Eira J, Lopes AT (2014). Peripheral nervous system plasmalogens regulate Schwann cell differentiation and myelination. J Clin Invest.

[CR57] Dahabieh MS, Ha Z, Di Pietro E (2017). Peroxisomes protect lymphoma cells from HDAC inhibitor-mediated apoptosis. Cell Death Differ.

[CR58] David C, Koch J, Oeljeklaus S (2013). A combined approach of quantitative interaction proteomics and live-cell imaging reveals a regulatory role for endoplasmic reticulum (ER) reticulon homology proteins in peroxisome biogenesis. Mol Cell Proteom.

[CR59] Dawidowski M, Emmanouilidis L, Kalel VC (2017). Inhibitors of PEX14 disrupt protein import into glycosomes and kill *Trypanosoma* parasites. Science.

[CR60] De Duve C (1965). Functions of microbodies (peroxisomes). J Cell Biol.

[CR61] De Duve C, Baudhuin P (1966). Peroxisomes (microbodies and related particles). Physiol Rev.

[CR62] De Vos KJ, Hafezparast M (2017). Neurobiology of axonal transport defects in motor neuron diseases: Opportunities for translational research?. Neurobiol Dis.

[CR63] De Munter S, Bamps D, Malheiro AR (2018). Autonomous Purkinje cell axonal dystrophy causes ataxia in peroxisomal multifunctional protein-2 deficiency. Brain Pathol.

[CR64] Delille HK, Agricola B, Guimaraes SC (2010). Pex11pbeta-mediated growth and division of mammalian peroxisomes follows a maturation pathway. J Cell Sci.

[CR65] Delmaghani S, Defourny J, Aghaie A (2015). Hypervulnerability to sound exposure through impaired adaptive proliferation of peroxisomes. Cell.

[CR66] Deori NM, Kale A, Maurya PK, Nagotu S (2018). Peroxisomes: role in cellular ageing and age related disorders. Biogerontology.

[CR67] Di Cara F, Sheshachalam A, Braverman NE (2017). Peroxisome-mediated metabolism is required for immune response to microbial infection. Immunity.

[CR68] Dias AF, Rodrigues TA, Pedrosa AG (2017). The peroxisomal matrix protein translocon is a large cavity-forming protein assembly into which PEX5 protein enters to release its cargo. J Biol Chem.

[CR69] Dixit E, Boulant S, Zhang Y (2010). Peroxisomes are signaling platforms for antiviral innate immunity. Cell.

[CR70] Dorninger F, Forss-Petter S, Berger J (2017). From peroxisomal disorders to common neurodegenerative diseases—the role of ether phospholipids in the nervous system. FEBS Lett.

[CR71] Dorninger F, Moser AB, Kou J (2018). Alterations in the plasma levels of specific choline phospholipids in Alzheimer’s disease mimic accelerated aging. J Alzheimers Dis.

[CR72] Dowling DK, Simmons LW (2009). Reactive oxygen species as universal constraints in life-history evolution. Proc Biol Sci.

[CR73] Du X, Brown AJ, Yang H (2015). Novel mechanisms of intracellular cholesterol transport: oxysterol-binding proteins and membrane contact sites. Curr Opin Cell Biol.

[CR74] Eastmond PJ (2007). Monodehydroascorbate reductase 4 is required for seed storage, oil hydrolysis and postgerminative growth in Arabidopsis. Plant Cell.

[CR75] Ebberink MS, Koster J, Visser G (2012). A novel defect of peroxisome division due to a homozygous non-sense mutation in the PEX11β gene. J Med Genet.

[CR76] Eberhart T, Kovacs W (2018). Pexophagy in yeast and mammals: an update on mysteries. Histochem Cell Biol.

[CR77] Effelsberg D, Cruz-Zaragoza LD, Schliebs W, Erdmann R (2016). Pex9p is a new yeast peroxisomal import receptor for PTS1-containing proteins. J Cell Sci.

[CR78] Elbaz-Alon Y, Morgan B, Clancy A et al (2014) The yeast oligopeptide transporter Opt2 is localized to peroxisomes and affects glutathione redox homeostasis. FEMS Yeast Res 1–13. 10.1111/1567-1364.12196 10.1111/1567-1364.12196 25130273

[CR79] Elsner M, Gehrmann W, Lenzen S (2011). Peroxisome-generated hydrogen peroxide as important mediator of lipotoxicity in insulin-producing cells. Diabetes.

[CR80] Engelen M, Kemp S, Poll-The B-T (2014). X-Linked adrenoleukodystrophy: pathogenesis and treatment. Curr Neurol Neurosci Rep.

[CR81] Erdmann R (2016). Assembly, maintenance and dynamics of peroxisomes. Biochim Biophys Acta Mol Cell Res.

[CR82] Fahrner JA, Liu R, Perry MS (2016). A novel de novo dominant negative mutation in DNM1L impairs mitochondrial fission and presents as childhood epileptic encephalopathy. Am J Med Genet A.

[CR83] Fan C, Pan J, Usuda N (1998). Steatohepatitis, spontaneous peroxisome proliferation and liver tumors in mice lacking peroxisomal fatty acyl-CoA oxidase. J Biol Chem.

[CR84] Fan J, Li X, Issop L (2016). ACBD2/ECI2-mediated peroxisome–mitochondria interactions in leydig cell steroid biosynthesis. Mol Endocrinol.

[CR85] Fanelli F, Sepe S, D’Amelio M (2013). Age-dependent roles of peroxisomes in the hippocampus of a transgenic mouse model of Alzheimer’s disease. Mol Neurodegener.

[CR86] Farré J-C, Carolino K, Stasyk OV (2017). A new yeast peroxin, Pex36, a functional homolog of mammalian PEX16, functions in the ER-to-peroxisome traffic of peroxisomal membrane proteins. J Mol Biol.

[CR87] Faust PL, Kovacs WJ (2014). Cholesterol biosynthesis and ER stress in peroxisome deficiency. Biochimie.

[CR88] Ferdinandusse S, Denis S, Clayton PT (2000). Mutations in the gene encoding peroxisomal α-methylacyl-CoA racemase cause adult-onset sensory motor neuropathy. Nat Genet.

[CR89] Ferdinandusse S, Denis S, Hogenhout EM (2007). Clinical, biochemical, and mutational spectrum of peroxisomal acyl-coenzyme A oxidase deficiency. Hum Mutat.

[CR90] Ferdinandusse S, Falkenberg KD, Koster J (2017). ACBD5 deficiency causes a defect in peroxisomal very long-chain fatty acid metabolism. J Med Genet.

[CR91] Ferdinandusse S, Denis S, van Roermund CWT (2018). A novel case of ACOX2 deficiency leads to recognition of a third human peroxisomal acyl-CoA oxidase. Biochim Biophys Acta.

[CR92] Fourcade S, Lopez-Erauskin J, Galino J (2008). Early oxidative damage underlying neurodegeneration in X-adrenoleukodystrophy. Hum Mol Genet.

[CR93] Fourcade S, López-Erauskin J, Ruiz M (2014). Mitochondrial dysfunction and oxidative damage cooperatively fuel axonal degeneration in X-linked adrenoleukodystrophy. Biochimie.

[CR94] Francisco T, Rodrigues TA, Dias AF (2017). Protein transport into peroxisomes: knowns and unknowns. BioEssays.

[CR95] Fransen M, Lismont C (2018). Redox signaling from and to peroxisomes: progress, challenges, and prospects. Antioxid Redox Signal.

[CR96] Fransen M, Nordgren M, Wang B, Apanasets O (2012). Role of peroxisomes in ROS/RNS-metabolism: Implications for human disease. Biochim Biophys Acta.

[CR97] Fransen M, Lismont C, Walton P (2017). The peroxisome–mitochondria connection: how and why?. Int J Mol Sci.

[CR98] Frederiks WM, Bosch KS, Hoeben KA (2010). Renal cell carcinoma and oxidative stress: the lack of peroxisomes. Acta Histochem.

[CR99] Frick EM, Strader LC (2018). Kinase MPK17 and the peroxisome division factor PMD1 influence salt-induced peroxisome proliferation. Plant Physiol.

[CR100] Friedman JR, Lackner LL, West M (2011). ER tubules mark sites of mitochondrial division. Science.

[CR101] Fujiki Y, Miyata N, Mukai S (2017). BAK regulates catalase release from peroxisomes. Mol Cell Oncol.

[CR102] Fujino T, Yamada T, Asada T (2017). Efficacy and blood plasmalogen changes by oral Administration Of Plasmalogen In Patients With Mild Alzheimer’s disease and mild cognitive impairment: a multicenter, randomized, double-blind, placebo-controlled trial. EBioMedicine.

[CR103] Galiani S, Waithe D, Reglinski K (2016). Super-resolution microscopy reveals compartmentalization of peroxisomal membrane proteins. J Biol Chem.

[CR104] Gandre-Babbe S, van der Bliek AM (2008). The novel tail-anchored membrane protein Mff controls mitochondrial and peroxisomal fission in mammalian cells. Mol Biol Cell.

[CR105] Gardner BM, Castanzo DT, Chowdhury S (2018). The peroxisomal AAA-ATPase Pex1/Pex6 unfolds substrates by processive threading. Nat Commun.

[CR106] Gaunt GL, de Duve C (1976). Subcellular distribution of D-amino acid oxidase and catalase in rat brain. J Neurochem.

[CR107] Gerber S, Charif M, Chevrollier A (2017). Mutations in DNM1L, as in OPA1, result in dominant optic atrophy despite opposite effects on mitochondrial fusion and fission. Brain.

[CR108] Ghaffari P, Mardinoglu A, Nielsen J (2015). Cancer metabolism: a modeling perspective. Front Physiol.

[CR109] Giordano CR, Terlecky SR (2012). Peroxisomes, cell senescence, and rates of aging. BBA Mol Basis Dis.

[CR110] Glasauer A, Chandel NS (2014). Targeting antioxidants for cancer therapy. Biochem Pharmacol.

[CR111] Goepfert S, Poirier Y (2007). Beta-oxidation in fatty acid degradation and beyond. Curr Opin Plant Biol.

[CR112] Goldfischer S, Moore CL, Johnson AB (1973). Peroxisomal and mitochondrial defects in the cerebro-hepato-renal syndrome. Science.

[CR113] Gray E, Rice C, Hares K (2014). Reductions in neuronal peroxisomes in multiple sclerosis grey matter. Mult Scler.

[CR114] Griffiths I, Klugmann M, Anderson T (1998). Axonal swellings and degeneration in mice lacking the major proteolipid of myelin. Science.

[CR115] Guimaraes SC, Schuster M, Bielska E (2015). Peroxisomes, lipid droplets, and endoplasmic reticulum “hitchhike” on motile early endosomes. J Cell Biol.

[CR116] Hayashi Y, Hayashi M, Hayashi H (2001). Direct interaction between glyoxysomes and lipid bodies in cotyledons of the *Arabidopsis thaliana* ped1 mutant. Protoplasma.

[CR117] Hein S, Schönfeld P, Kahlert S, Reiser G (2008). Toxic effects of X-linked adrenoleukodystrophy-associated, very long chain fatty acids on glial cells and neurons from rat hippocampus in culture. Hum Mol Genet.

[CR118] Helle SCJ, Feng Q, Aebersold MJ (2017). Mechanical force induces mitochondrial fission. Elife.

[CR119] Herbig U, Ferreira M, Condel L (2006). Cellular senescence in aging primates. Science.

[CR120] Herzog K, Pras-Raves ML, Ferdinandusse S (2017). Functional characterisation of peroxisomal β-oxidation disorders in fibroblasts using lipidomics. J Inherit Metab Dis doi.

[CR121] Hettema EH, Gould SJ (2017). Cell biology: Organelle formation from scratch. Nature.

[CR122] Hettema EH, Erdmann R, van der Klei IJ, Veenhuis M (2014). Evolving models for peroxisome biogenesis. Curr Opin Cell Biol.

[CR123] Heymans HS, Schutgens RB, Tan R (1983). Severe plasmalogen deficiency in tissues of infants without peroxisomes (Zellweger syndrome). Nature.

[CR124] Hoepfner D, van den Berg M, Philippsen P (2001). A role for Vps1p, actin, and the Myo2p motor in peroxisome abundance and inheritance in *Saccharomyces cerevisiae*. J Cell Biol.

[CR125] Hoepfner D, Schildknegt D, Braakman I (2005). Contribution of the endoplasmic reticulum to peroxisome formation. Cell.

[CR126] Horner SM, Wilkins C, Badil S (2015). Proteomic analysis of mitochondrial-associated ER membranes (MAM) during RNA virus infection reveals dynamic changes in protein and organelle trafficking. PLoS One.

[CR127] Horton JD, Goldstein JL, Brown MS (2002). SREBPs: activators of the complete program of cholesterol and fatty acid synthesis in the liver. J Clin Invest.

[CR128] Hossain MS, Abe Y, Ali F (2017). Reduction of ether-type glycerophospholipids, plasmalogens, by NF-κB signal leading to microglial activation. J Neurosci.

[CR129] Hruban Z, Rechcigl M (1969). Microbodies and related particles. Morphology, biochemistry, and physiology. Int Rev Cytol Suppl.

[CR130] Hu J (2010). Molecular basis of peroxisome division and proliferation in plants. Int Rev Cell Mol Biol.

[CR131] Hua R, Cheng D, Coyaud É (2017). VAPs and ACBD5 tether peroxisomes to the ER for peroxisome maintenance and lipid homeostasis. J Cell Biol.

[CR132] Huang J, Viswakarma N, Yu S (2011). Progressive endoplasmic reticulum stress contributes to hepatocarcinogenesis in fatty acyl-CoA oxidase 1-deficient mice. Am J Pathol.

[CR133] Huber N, Guimaraes S, Schrader M (2013). Charcot-Marie-Tooth disease-associated mutants of GDAP1 dissociate its roles in peroxisomal and mitochondrial fission. EMBO Rep.

[CR134] Hulshagen L, Krysko O, Bottelbergs A (2008). Absence of functional peroxisomes from mouse CNS causes dysmyelination and axon degeneration. J Neurosci.

[CR135] Huybrechts SJ, Van Veldhoven PP, Brees C (2009). Peroxisome dynamics in cultured mammalian cells. Traffic.

[CR136] Huyghe S, Schmalbruch H, Hulshagen L (2006). Peroxisomal multifunctional protein-2 deficiency causes motor deficits and glial lesions in the adult central nervous system. Am J Pathol.

[CR137] Inestrosa NC, Carvajal FJ, Zolezzi JM (2013). Peroxisome proliferators reduce spatial memory impairment, synaptic failure, and neurodegeneration in brains of a double transgenic mice model of Alzheimer’s disease. J Alzheimers Dis.

[CR138] Islinger M, Li KW, Loos M (2010). Peroxisomes from the heavy mitochondrial fraction: isolation by zonal free flow electrophoresis and quantitative mass spectrometrical characterization. J Proteome Res.

[CR139] Islinger M, Cardoso MJR, Schrader M (2010). Be different—the diversity of peroxisomes in the animal kingdom. Biochim Biophys Acta.

[CR140] Islinger M, Grille S, Fahimi HD, Schrader M (2012). The peroxisome: an update on mysteries. Histochem Cell Biol.

[CR141] Islinger M, Abdolzade-bavil A, Liebler S, Josic D, Hixson DC (2012). Assessing heterogeneity of peroxisomes: isolation of two subpopulations from rat liver. Liver Proteomics: methods and protocols.

[CR142] Itoyama A, Michiyuki S, Honsho M (2013). Mff functions with Pex11p and DLP1 in peroxisomal fission. Biol Open.

[CR143] Ježek J, Cooper K, Strich R (2018). Reactive oxygen species and mitochondrial dynamics: the Yin and Yang of mitochondrial dysfunction and cancer progression. Antioxidants.

[CR144] Jiang Z, Woda BA, Rock KL (2001). P504S: a new molecular marker for the detection of prostate carcinoma. Am J Surg Pathol.

[CR145] Jiang Z, Fanger GR, Banner BF (2003). A dietary enzyme: alpha-methylacyl-CoA racemase/P504S is overexpressed in colon carcinoma. Cancer Detect Prev.

[CR146] Jindal Y, Singh A, Kumar R (2016). Expression of alpha methylacyl CoA racemase (AMACR) in gastric adenocarcinoma and its correlation with helicobacter pylori infection. J Clin Diagn Res.

[CR147] Joshi AS, Huang X, Choudhary V (2016). A family of membrane-shaping proteins at ER subdomains regulates pre-peroxisomal vesicle biogenesis. J Cell Biol.

[CR148] Kagan JC (2012). Signaling organelles of the innate immune system. Cell.

[CR149] Kassmann CM, Lappe-Siefke C, Baes M (2007). Axonal loss and neuroinflammation caused by peroxisome-deficient oligodendrocytes. Nat Genet.

[CR150] Kassmann CM, Quintes S, Rietdorf J (2011). A role for myelin-associated peroxisomes in maintaining paranodal loops and axonal integrity. FEBS Lett.

[CR151] Kataria Y, Wright M, Deaton RJ (2015). Dietary influences on tissue concentrations of phytanic acid and AMACR expression in the benign human prostate. Prostate.

[CR152] Kazmierczak M, Kazmierczak P, Peng AW (2017). Pejvakin, a candidate stereociliary rootlet protein, regulates hair cell function in a cell-autonomous manner. J Neurosci.

[CR153] Keller JM, Cablé S, el Bouhtoury F (1993). Peroxisome through cell differentiation and neoplasia. Biol Cell.

[CR154] Kleinecke S, Richert S, de Hoz L (2017). Peroxisomal dysfunctions cause lysosomal storage and axonal Kv1 channel redistribution in peripheral neuropathy. Elife.

[CR155] Knoblach B, Rachubinski R (2015). Sharing the cell’s bounty—organelle inheritance in yeast. J Cell Sci.

[CR156] Knoblach B, Rachubinski RA (2016). How peroxisomes partition between cells. A story of yeast, mammals and filamentous fungi. Curr Opin Cell Biol.

[CR157] Knoblach B, Sun X, Coquelle N (2013). An ER-peroxisome tether exerts peroxisome population control in yeast. EMBO J.

[CR158] Knoops K, Manivannan S, Cepinska MN (2014). Preperoxisomal vesicles can form in the absence of Pex3. J Cell Biol.

[CR159] Koch J, Brocard C (2012). PEX11 proteins attract Mff and hFis1 to coordinate peroxisomal fission. J Cell Sci.

[CR160] Koch A, Thiemann M, Grabenbauer M (2003). Dynamin-like protein 1 is involved in peroxisomal fission. J Biol Chem.

[CR161] Koch A, Yoon Y, Bonekamp NA (2005). A role for Fis1 in both mitochondrial and peroxisomal fission in mammalian cells. Mol Biol Cell.

[CR162] Koch J, Feichtinger RG, Freisinger P (2016). Disturbed mitochondrial and peroxisomal dynamics due to loss of MFF causes Leigh-like encephalopathy, optic atrophy and peripheral neuropathy. J Med Genet.

[CR163] Kou J, Kovacs GG, Höftberger R (2011). Peroxisomal alterations in Alzheimer’s disease. Acta Neuropathol.

[CR164] Kovacs WJ, Olivier LM, Krisans SK (2002). Central role of peroxisomes in isoprenoid biosynthesis. Prog Lipid Res.

[CR165] Kovacs WJ, Tape KN, Shackelford JE (2009). Peroxisome deficiency causes a complex phenotype because of hepatic SREBP/Insig dysregulation associated with endoplasmic reticulum stress. J Biol Chem.

[CR166] Krahling JB, Gee R, Gauger JA, Tolbert NE (1979). Postnatal development of peroxisomal and mitochondrial enzymes in rat liver. J Cell Physiol.

[CR167] Kruska N, Schönfeld P, Pujol A, Reiser G (2015). Astrocytes and mitochondria from adrenoleukodystrophy protein (ABCD1)-deficient mice reveal that the adrenoleukodystrophy-associated very long-chain fatty acids target several cellular energy-dependent functions. Biochim Biophys Acta.

[CR168] Krysko O, Hulshagen L, Janssen A (2007). Neocortical and cerebellar developmental abnormalities in conditions of selective elimination of peroxisomes from brain or from liver. J Neurosci Res.

[CR169] Kumar S, de Boer R, van der Klei IJ (2018). Yeast cells contain a heterogeneous population of peroxisomes that segregate asymmetrically during cell division. J Cell Sci.

[CR170] Kuravi K, Nagotu S, Krikken AM (2006). Dynamin-related proteins Vps1p and Dnm1p control peroxisome abundance in *Saccharomyces cerevisiae*. J Cell Sci.

[CR171] Lauer C, Völkl A, Riedl S (1999). Impairment of peroxisomal biogenesis in human colon carcinoma. Carcinogenesis.

[CR172] Laurenti G, Benedetti E, D’Angelo B (2011). Hypoxia induces peroxisome proliferator-activated receptor ?? (PPAR??) and lipid metabolism peroxisomal enzymes in human glioblastoma cells. J Cell Biochem.

[CR173] Lenzen S, Drinkgern J, Tiedge M (1996). Low antioxidant enzyme gene expression in pancreatic islets compared with various other mouse tissues. Free Radic Biol Med.

[CR174] Lewis SC, Uchiyama LF, Nunnari J (2016). ER–mitochondria contacts couple mtDNA synthesis with mitochondrial division in human cells. Science.

[CR175] Li X, Gould SJ (2003). The dynamin-like GTPase DLP1 is essential for peroxisome division and is recruited to peroxisomes in part by PEX11. J Biol Chem.

[CR176] Liegel RP, Ronchetti A, Sidjanin DJ (2014). Alkylglycerone phosphate synthase (AGPS) deficient mice: Models for rhizomelic chondrodysplasia punctata type 3 (RCDP3) malformation syndrome. Mol Genet Metab Reports.

[CR177] Lin C, Steinberg G (2017). Spatial organization of organelles in fungi: insights from mathematical modelling. Fungal Genet Biol.

[CR178] Lin C, Schuster M, Guimaraes SC (2016). Active diffusion and microtubule-based transport oppose myosin forces to position organelles in cells. Nat Commun.

[CR179] Lingwood D, Simons K (2010). Lipid rafts as a membrane-organizing principle. Science.

[CR180] Lismont C, Nordgren M, Van Veldhoven PP, Fransen M (2015). Redox interplay between mitochondria and peroxisomes. Front Cell Dev Biol.

[CR181] Litwin JA, Beier K, Völkl A (1999). Immunocytochemical investigation of catalase and peroxisomal lipid beta-oxidation enzymes in human hepatocellular tumors and liver cirrhosis. Virchows Arch.

[CR182] Lloyd MD, Darley DJ, Wierzbicki AS, Threadgill MD (2008). Alpha-methylacyl-CoA racemase—an “obscure” metabolic enzyme takes centre stage. FEBS J.

[CR183] López-Erauskin J, Galino J, Ruiz M (2013). Impaired mitochondrial oxidative phosphorylation in the peroxisomal disease X-linked adrenoleukodystrophy. Hum Mol Genet.

[CR184] Lu T, Finkel T (2008). Free radicals and senescence. Exp Cell Res.

[CR185] Lucki I (1998). The spectrum of behaviors influenced by serotonin. Biol Psychiatry.

[CR186] Lüers G, Hashimoto T, Fahimi HD, Völkl A (1993). Biogenesis of peroxisomes: isolation and characterization of two distinct peroxisomal populations from normal and regenerating rat liver. J Cell Biol.

[CR187] Marcassa E, Kallinos A, Jardine J (2018). Dual role of USP30 in controlling basal pexophagy and mitophagy. EMBO Rep.

[CR188] Martinez-Vicente M (2017). Neuronal mitophagy in neurodegenerative diseases. Front Mol Neurosci.

[CR189] Mast FD, Rachubinski RA, Aitchison JD (2015). Signaling dynamics and peroxisomes. Curr Opin Cell Biol.

[CR190] Mast FD, Jamakhandi A, Saleem RA (2016). Peroxins Pex30 and Pex29 dynamically associate with reticulons to regulate peroxisome biogenesis from the endoplasmic reticulum. J Biol Chem.

[CR191] Mast FD, Herricks T, Strehler KM (2018). ESCRT-III is required for scissioning new peroxisomes from the endoplasmic reticulum. J Cell Biol.

[CR192] Mattiazzi Ušaj M, Brložnik M, Kaferle P (2015). Genome-wide localization study of yeast Pex11 identifies peroxisome–mitochondria interactions through the ERMES complex. J Mol Biol.

[CR193] Maxfield FR, Wüstner D (2002). Intracellular cholesterol transport. J Clin Invest.

[CR194] McClelland GB, Khanna S, González GF (2003). Peroxisomal membrane monocarboxylate transporters: evidence for a redox shuttle system?. Biochem Biophys Res Commun.

[CR195] Meinecke M, Cizmowski C, Schliebs W (2010). The peroxisomal importomer constitutes a large and highly dynamic pore. Nat Cell Biol.

[CR196] Mesquita A, Weinberger M, Silva A (2010). Caloric restriction or catalase inactivation extends yeast chronological lifespan by inducing H2O2 and superoxide dismutase activity. Proc Natl Acad Sci USA.

[CR197] Monte MJ, Alonso-Peña M, Briz O (2017). ACOX2 deficiency: an inborn error of bile acid synthesis identified in an adolescent with persistent hypertransaminasemia. J Hepatol.

[CR198] Montilla-Martinez M, Beck S, Klümper J (2015). Distinct pores for peroxisomal import of PTS1 and PTS2 proteins. Cell Rep.

[CR199] Motley AM, Hettema EH (2007). Yeast peroxisomes multiply by growth and division. J Cell Biol.

[CR200] Motley AM, Ward GP, Hettema EH (2008). Dnm1p-dependent peroxisome fission requires Caf4p, Mdv1p and Fis1p. J Cell Sci.

[CR201] Müller CC, Nguyen TH, Ahlemeyer B (2011). PEX13 deficiency in mouse brain as a model of Zellweger syndrome: abnormal cerebellum formation, reactive gliosis and oxidative stress. Dis Model Mech.

[CR202] Nagotu S, Saraya R, Otzen M (2008). Peroxisome proliferation in Hansenula polymorpha requires Dnm1p which mediates fission but not de novo formation. Biochim Biophys Acta.

[CR203] Narendra S, Venkataramani S, Shen G (2006). The Arabidopsis ascorbate peroxidase 3 is a peroxisomal membrane-bound antioxidant enzyme and is dispensable for Arabidopsis growth and development. J Exp Bot.

[CR204] Nasca A, Legati A, Baruffini E (2016). Biallelic mutations in DNM1L are associated with a slowly progressive infantile encephalopathy. Hum Mutat.

[CR205] Nath N, Khan M, Rattan R (2009). Loss of AMPK exacerbates experimental autoimmune encephalomyelitis disease severity. Biochem Biophys Res Commun.

[CR206] Nazarko TY, Ozeki K, Till A (2014). Peroxisomal Atg37 binds Atg30 or palmitoyl-CoA to regulate phagophore formation during pexophagy. J Cell Biol.

[CR207] Neill S, Barros R, Bright J (2008). Nitric oxide, stomatal closure, and abiotic stress. J Exp Bot.

[CR208] Neuhaus A, Eggeling C, Erdmann R, Schliebs W (2016). Why do peroxisomes associate with the cytoskeleton?. Biochim Biophys Acta Mol Cell Res.

[CR209] Neuspiel M, Schauss AC, Braschi E (2008). Cargo-selected transport from the mitochondria to peroxisomes is mediated by vesicular carriers. Curr Biol.

[CR210] Nury T, Zarrouk A, Ragot K (2017). 7-Ketocholesterol is increased in the plasma of X-ALD patients and induces peroxisomal modifications in microglial cells: Potential roles of 7-ketocholesterol in the pathophysiology of X-ALD. J Steroid Biochem Mol Biol.

[CR211] Ogata T, Iijima S, Hoshikawa S (2004). Opposing extracellular signal-regulated kinase and Akt pathways control Schwann cell myelination. J Neurosci.

[CR212] Okumoto K, Ono T, Toyama R (2018). New splicing variants of mitochondrial Rho GTPase-1 (Miro1) transport peroxisomes. J Cell Biol.

[CR213] Opaliński L, Kiel JAKW, Williams C (2011). Membrane curvature during peroxisome fission requires Pex11. EMBO J.

[CR214] Paintlia AS, Paintlia MK, Singh AK, Singh I (2013). Modulation of Rho-Rock signaling pathway protects oligodendrocytes against cytokine toxicity via PPAR-α-dependent mechanism. Glia.

[CR215] Pascual-Ahuir A, Manzanares-Estreder S, Proft M (2017). Pro- and antioxidant functions of the peroxisome–mitochondria connection and its impact on aging and disease. Oxid Med Cell Longev.

[CR216] Pedrosa AG, Francisco T, Bicho D (2018). Peroxisomal monoubiquitinated PEX5 interacts with the AAA ATPases PEX1 and PEX6 and is unfolded during its dislocation into the cytosol. JBC.

[CR217] Piano V, Benjamin DI, Valente S (2015). Discovery of inhibitors for the ether lipid-generating enzyme AGPS as anti-cancer agents. ACS Chem Biol.

[CR218] Pihl-Jensen G, Tsakiri A, Frederiksen JL (2015). Statin treatment in multiple sclerosis: a systematic review and meta-analysis. CNS Drugs.

[CR219] Pljesa-Ercegovac M, Mimic-Oka J, Dragicevic D (2008). Altered antioxidant capacity in human renal cell carcinoma: role of glutathione associated enzymes. Urol Oncol.

[CR220] Prasad SR, Narra VR, Shah R (2007). Segmental disorders of the nephron: histopathological and imaging perspective. Br J Radiol.

[CR221] Rahim RS, Meedeniya ACB, Crane DI (2014). Central serotonergic neuron deficiency in a mouse model of Zellweger syndrome. Neuroscience.

[CR222] Rahim RS, Chen M, Nourse CC (2016). Mitochondrial changes and oxidative stress in a mouse model of Zellweger syndrome neuropathogenesis. Neuroscience.

[CR223] Raychaudhuri S, Prinz WA (2008). Nonvesicular phospholipid transfer between peroxisomes and the endoplasmic reticulum. Proc Natl Acad Sci USA.

[CR224] Renquist BJ, Madanayake TW, Ghimire S (2018). Transmembrane protein 135 (TMEM135) is a liver X receptor target gene that mediates an auxiliary peroxisome matrix protein import pathway. bioRxiv.

[CR225] Rhodin J (1954) Correlation of ultrastructural organization and function in normal and experimentally changed proximal convoluted tubule cells of the mouse kidney. Thesis, Karolinska Institutet, Aktiebolaget Godvil, Stockholm

[CR226] Rodemer C, Thai T, Brugger B (2003). Inactivation of ether lipid biosynthesis causes male infertility, defects in eye development and optic nerve hypoplasia in mice. Hum Mol Genet.

[CR227] Rouiller C, Bernhard W (1956). Microbodies and the problem of mitochondrial regeneration in liver cells. J Biophys Biochem Cytol.

[CR228] Ruokun C, Yake X, Fengdong Y (2016). Lentivirus-mediated silencing of HSDL2 suppresses cell proliferation in human gliomas. Tumour Biol.

[CR229] Salmon AB, Richardson A, Pérez VI (2010). Update on the oxidative stress theory of aging: does oxidative stress play a role in aging or healthy aging?. Free Radic Biol Med.

[CR230] Salogiannis J, Reck-Peterson SL (2017). Hitchhiking: a non-canonical mode of microtubule-based transport. Trends Cell Biol.

[CR231] Salogiannis J, Egan MJ, Reck-Peterson SL (2016). Peroxisomes move by hitchhiking on early endosomes using the novel linker protein PxdA. J Cell Biol.

[CR232] Saraya R, Veenhuis M, Klei IJ, Van Der (2010). Peroxisomes as dynamic organelles: peroxisome abundance in yeast. FEBS J.

[CR233] Scherz-Shouval R, Elazar Z (2011). Regulation of autophagy by ROS: physiology and pathology. Trends Biochem Sci.

[CR234] Schönfeld P, Struy H (1999). Refsum disease diagnostic marker phytanic acid alters the physical state of membrane proteins of liver mitochondria. FEBS Lett.

[CR235] Schrader M (2001). Tubulo—reticular clusters of peroxisomes in living COS-7 cells: dynamic behavior and association with lipid droplets. J Histochem Cytochem.

[CR236] Schrader M, Fahimi HD (2008). The peroxisome: still a mysterious organelle. Histochem Cell Biol.

[CR237] Schrader M, Pellegrini L (2017). The making of a mammalian peroxisome, version 2.0: mitochondria get into the mix. Cell Death Differ.

[CR238] Schrader M, Baumgart E, Volkl A, Fahimi HD (1994). Heterogeneity of peroxisomes in human hepatoblastoma cell line HepG2. Evidence of distinct subpopulations. Eur J Cell Biol.

[CR239] Schrader M, Thiemann M, Fahimi HD (2003). Peroxisomal motility and interaction with microtubules. Microsc Res Tech.

[CR240] Schrader M, Grille S, Fahimi HD, Islinger M (2013). Peroxisome interactions and cross-talk with other subcellular compartments in animal cells. Subcell Biochem.

[CR241] Schrader M, Costello J, Godinho LF, Islinger M (2015). Peroxisome–mitochondria interplay and disease. J Inherit Metab Dis.

[CR242] Schrader M, Godinho LF, Costello JL, Islinger M (2015). The different facets of organelle interplay—an overview of organelle interactions. Front Cell Dev Biol.

[CR243] Schrader M, Costello JL, Godinho LF (2016). Proliferation and fission of peroxisomes—an update. Biochim Biophys Acta Mol Cell Res.

[CR244] Schwerter DP, Grimm I, Platta HW, Erdmann R (2017). ATP-driven processes of peroxisomal matrix protein import. Biol Chem.

[CR245] Shai N, Schuldiner M, Zalckvar E (2016). No peroxisome is an island—peroxisome contact sites. Biochim Biophys Acta Mol Cell Res.

[CR246] Shai N, Yifrach E, van Roermund CWT (2018). Systematic mapping of contact sites reveals tethers and a function for the peroxisome–mitochondria contact. Nat Commun.

[CR247] Shamseldin HE, Alshammari M, Al-Sheddi T (2012). Genomic analysis of mitochondrial diseases in a consanguineous population reveals novel candidate disease genes. J Med Genet.

[CR248] Sharon R, Bar-Joseph I, Frosch MP (2003). The formation of highly soluble oligomers of alpha-synuclein is regulated by fatty acids and enhanced in Parkinson’s disease. Neuron.

[CR249] Sheffer R, Douiev L, Edvardson S (2016). Postnatal microcephaly and pain insensitivity due to a de novo heterozygous DNM1L mutation causing impaired mitochondrial fission and function. Am J Med Genet A.

[CR250] Shi R, Zhang Y, Shi Y (2012). Inhibition of peroxisomal β-oxidation by thioridazine increases the amount of VLCFAs and Aβ generation in the rat brain. Neurosci Lett.

[CR251] Singh I, Paintlia AS, Khan M (2004). Impaired peroxisomal function in the central nervous system with inflammatory disease of experimental autoimmune encephalomyelitis animals and protection by lovastatin treatment. Brain Res.

[CR252] Singh I, Samuvel DJ, Choi S (2018). Combination therapy of lovastatin and AMP-activated protein kinase activator improves mitochondrial and peroxisomal functions and clinical disease in experimental autoimmune encephalomyelitis model. Immunology.

[CR253] Sleat DE, Wiseman JA, El-Banna M (2004). Genetic evidence for nonredundant functional cooperativity between NPC1 and NPC2 in lipid transport. Proc Natl Acad Sci USA.

[CR254] Soliman K, Göttfert F, Rosewich H (2018). Super-resolution imaging reveals the sub-diffraction phenotype of Zellweger Syndrome ghosts and wild-type peroxisomes. Sci Rep.

[CR255] Sparkes I, Gao H (2014). Plant peroxisome dynamics: movement, positioning and connections. Molecular machines involved in peroxisome biogenesis and maintenance.

[CR256] Spillantini MG, Schmidt ML, Lee VM-Y (1997). α-synuclein in Lewy bodies. Nature.

[CR257] Stanislaus R, Gilg AG, Singh AK, Singh I (2002). Immunomodulation of experimental autoimmune encephalomyelitis in the Lewis rats by Lovastatin. Neurosci Lett.

[CR258] Steinberg G (2016). The mechanism of peroxisome motility in filamentous fungi. Fungal Genet Biol.

[CR259] Su J, Thomas AS, Grabietz T (2018). The N-terminal amphipathic helix of Pex11p self-interacts to induce membrane remodelling during peroxisome fission. Biochim Biophys Acta Biomembr.

[CR260] Sugiura A, Mattie S, Prudent J, McBride HM (2017). Newly born peroxisomes are a hybrid of mitochondrial and ER-derived pre-peroxisomes. Nature.

[CR261] Sun Q, Zhang Y, Su J (2018). Role of hydroxysteroid dehydrogenase-like 2 (HSDL2) in human ovarian cancer. Med Sci Monit.

[CR262] Takano-Rojas H, Zickler D, Peraza-Reyes L (2016). Peroxisome dynamics during development of the fungus Podospora anserina. Mycologia.

[CR263] Taylor JP, Brown RH, Cleveland DW (2016). Decoding ALS: from genes to mechanism. Nature.

[CR264] Taylor RL, Handley MT, Waller S (2017). Novel PEX11B mutations extend the peroxisome biogenesis disorder 14B phenotypic spectrum and underscore congenital cataract as an early feature. Invest Ophthalmol Vis Sci.

[CR265] Teigler A, Komljenovic D, Draguhn A (2009). Defects in myelination, paranode organization and Purkinje cell innervation in the ether lipid-deficient mouse cerebellum. Hum Mol Genet.

[CR266] Titorenko VI, Rachubinski RA (2004). The peroxisome: orchestrating important developmental decisions from inside the cell. J Cell Biol.

[CR267] Titorenko VI, Rachubinski RA (2014). Origin and spatiotemporal dynamics of the peroxisomal endomembrane system. Front Physiol.

[CR268] Toyama EQ, Herzig S, Courchet J (2016). Metabolism. AMP-activated protein kinase mediates mitochondrial fission in response to energy stress. Science.

[CR269] Valcarcel-Jimenez L, Gaude E, Torrano V (2017). Mitochondrial metabolism: Yin and Yang for tumor progression. Trends Endocrinol Metab.

[CR270] Valença I, Pértega-Gomes N, Vizcaino JR (2015). Localization of MCT2 at peroxisomes is associated with malignant transformation in prostate cancer. J Cell Mol Med.

[CR271] Valm AM, Cohen S, Legant WR (2017). Applying systems-level spectral imaging and analysis to reveal the organelle interactome. Nature.

[CR272] Vamecq J, Andreoletti P, Latruffe N (2014). The human peroxisome in health and disease: the story of an oddity becoming a vital organelle. Biochimie.

[CR273] Van Veldhoven PP (2010). Biochemistry and genetics of inherited disorders of peroxisomal fatty acid metabolism. J Lipid Res.

[CR274] Vanstone JR, Smith AM, McBride S (2016). DNM1L-related mitochondrial fission defect presenting as refractory epilepsy. Eur J Hum Genet.

[CR275] Verheijden S, Bottelbergs A, Krysko O (2013). Peroxisomal multifunctional protein-2 deficiency causes neuroinflammation and degeneration of Purkinje cells independent of very long chain fatty acid accumulation. Neurobiol Dis.

[CR276] Verheijden S, Beckers L, De Munter S (2014). Central nervous system pathology in MFP2 deficiency: Insights from general and conditional knockout mouse models. Biochimie.

[CR277] Vilarinho S, Sari S, Mazzacuva F (2016). ACOX2 deficiency: a disorder of bile acid synthesis with transaminase elevation, liver fibrosis, ataxia, and cognitive impairment. Proc Natl Acad Sci USA.

[CR278] Vollmer T, Key L, Durkalski V (2004). Oral simvastatin treatment in relapsing-remitting multiple sclerosis. Lancet.

[CR279] Walter KM, Schönenberger MJ, Trötzmüller M (2014). Hif-2α promotes degradation of mammalian peroxisomes by selective autophagy. Cell Metab.

[CR280] Walton PA, Brees C, Lismont C (2017). The peroxisomal import receptor PEX5 functions as a stress sensor, retaining catalase in the cytosol in times of oxidative stress. Biochim Biophys Acta.

[CR281] Wanders RJA (2014). Metabolic functions of peroxisomes in health and disease. Biochimie.

[CR282] Wanders RJA, Poll-The BT (2017). Role of peroxisomes in human lipid metabolism and its importance for neurological development. Neurosci Lett.

[CR283] Wanders RJA, Waterham HR (2006). Biochemistry of mammalian peroxisomes revisited. Annu Rev Biochem.

[CR284] Wanders RJA, Ferdinandusse S, Brites P, Kemp S (2010). Peroxisomes, lipid metabolism and lipotoxicity. Biochim Biophys Acta.

[CR285] Wanders RJA, Komen J, Ferdinandusse S (2011). Phytanic acid metabolism in health and disease. Biochim Biophys Acta.

[CR286] Wanders RJA, Waterham HR, Ferdinandusse S (2016). Metabolic interplay between peroxisomes and other subcellular organelles including mitochondria and the endoplasmic reticulum. Front Cell Dev Biol Front Cell Dev Biol.

[CR287] Waterham HR, Koster J, van Roermund CWT (2007). A lethal defect of mitochondrial and peroxisomal fission. N Engl J Med.

[CR288] Weir HJ, Yao P, Huynh FK (2017). Dietary restriction and AMPK increase lifespan via mitochondrial network and peroxisome remodeling. Cell Metab.

[CR289] Weir NR, Kamber RA, Martenson JS, Denic V (2017). The AAA protein Msp1 mediates clearance of excess tail-anchored proteins from the peroxisomal membrane. Elife.

[CR290] Went PT, Sauter G, Oberholzer M, Bubendorf L (2006). Abundant expression of AMACR in many distinct tumour types. Pathology.

[CR291] Williams C, Opalinski L, Landgraf C (2015). The membrane remodeling protein Pex11p activates the GTPase Dnm1p during peroxisomal fission. Proc Natl Acad Sci USA.

[CR292] Witkiewicz AK, Varambally S, Shen R (2005). Alpha-methylacyl-CoA racemase protein expression is associated with the degree of differentiation in breast cancer using quantitative image analysis. Cancer Epidemiol Biomark Prev.

[CR293] Wong CP, Xu Z, Power C, Hobman TC (2018). Targeted elimination of peroxisomes during viral infection: lessons from HIV and other viruses. DNA Cell Biol.

[CR294] Wróblewska JP, Cruz-Zaragoza LD, Yuan W (2017). *Saccharomyces cerevisiae* cells lacking Pex3 contain membrane vesicles that harbor a subset of peroxisomal membrane proteins. Biochim Biophys Acta.

[CR295] Wu M, Ho S-M (2004). PMP24, a gene identified by MSRF, undergoes DNA hypermethylation-associated gene silencing during cancer progression in an LNCaP model. Oncogene.

[CR296] Wu X, Daniels G, Lee P, Monaco ME (2014). Lipid metabolism in prostate cancer. Am J Clin Exp Urol.

[CR297] Wu W, Liu F, Wu K (2018). Lon peptidase 2, peroxisomal (LONP2) contributes to cervical carcinogenesis via oxidative stress. Med Sci Monit.

[CR298] Wurmbach E, Chen Y, Khitrov G (2007). Genome-wide molecular profiles of HCV-induced dysplasia and hepatocellular carcinoma. Hepatology.

[CR299] Xiang C, Wang Y, Zhang H, Han F (2017). The role of endoplasmic reticulum stress in neurodegenerative disease. Apoptosis.

[CR300] Xu J, Thornburg T, Turner AR (2005). Serum levels of phytanic acid are associated with prostate cancer risk. Prostate.

[CR301] Xu Z, Asahchop EL, Branton WG (2017). MicroRNAs upregulated during HIV infection target peroxisome biogenesis factors: implications for virus biology, disease mechanisms and neuropathology. PLOS Pathog.

[CR302] Yagita Y, Hiromasa T, Fujiki Y (2013). Tail-anchored PEX26 targets peroxisomes via a PEX19-dependent and TRC40-independent class I pathway. J Cell Biol.

[CR303] Yagita Y, Shinohara K, Abe Y (2017). Deficiency of a retinal dystrophy protein, Acyl-CoA binding domain-containing 5 (ACBD5), impairs peroxisomal β-oxidation of very-long-chain fatty acids. J Biol Chem.

[CR304] Yakunin E, Moser A, Loeb V (2010). Alpha-Synuclein abnormalities in mouse models of peroxisome biogenesis disorders. J Neurosci Res.

[CR305] Yeagle P (1988). Biology of cholesterol.

[CR306] Yifrach E, Chuartzman SG, Dahan N (2016). Characterization of proteome dynamics during growth in oleate reveals a new peroxisome-targeting receptor. J Cell Sci.

[CR307] Yoboue ED, Sitia R, Simmen T (2018). Redox crosstalk at endoplasmic reticulum (ER) membrane contact sites (MCS) uses toxic waste to deliver messages. Cell Death Dis.

[CR308] Yofe I, Soliman K, Chuartzman SG (2017). Pex35 is a regulator of peroxisome abundance. J Cell Sci.

[CR309] Yoon G, Malam Z, Paton T (2016). Lethal disorder of mitochondrial fission caused by mutations in DNM1L. J Pediatr.

[CR310] Yoshida GJ (2015). Metabolic reprogramming: the emerging concept and associated therapeutic strategies. J Exp Clin Cancer Res.

[CR311] Yoshida Y, Niwa H, Honsho M (2015). Pex11mediates peroxisomal proliferation by promoting deformation of the lipid membrane. Biol Open.

[CR312] Young JM, Nelson JW, Cheng J (2015). Peroxisomal biogenesis in ischemic brain. Antioxid Redox Signal.

[CR313] Yu S, Matsusue K, Kashireddy P (2003). Adipocyte-specific gene expression and adipogenic steatosis in the mouse liver due to peroxisome proliferator-activated receptor γ1 (PPARγ1) overexpression. J Biol Chem.

[CR314] Zaha K, Matsumoto H, Itoh M (2016). DNM1L-related encephalopathy in infancy with Leigh syndrome-like phenotype and suppression-burst. Clin Genet.

[CR315] Žárský V, Doležal P (2016). Evolution of the Tim17 protein family. Biol Direct.

[CR316] Zentgraf U (2007). Oxidative stress and leaf senescence. Senescence processes in plants.

[CR317] Zha S, Ferdinandusse S, Denis S (2003). Alpha-methylacyl-CoA racemase as an androgen-independent growth modifier in prostate cancer. Cancer Res.

[CR318] Zha S, Ferdinandusse S, Hicks JL (2005). Peroxisomal branched chain fatty acid?—oxidation pathway is upregulated in prostate cancer. Prostate.

[CR319] Zhang X, Wu M, Xiao H (2010). Methylation of a single intronic CpG mediates expression silencing of the PMP24 gene in prostate cancer. Prostate.

[CR320] Zhang J, Kim J, Alexander A (2013). A tuberous sclerosis complex signalling node at the peroxisome regulates mTORC1 and autophagy in response to ROS. Nat Cell Biol.

[CR321] Zhou L, Yu M, Arshad M (2018). Coordination among lipid droplets, peroxisomes and mitochondria regulates energy expenditure through the CIDE-ATGL-PPARα pathway in adipocytes. Diabetes.

[CR322] Zimmermann P, Heinlein C, Orendi G, Zentgraf U (2006). Senescence-specific regulation of catalases in *Arabidopsis thaliana* (L.) Heynh. Plant Cell Environ.

